# Commercial Organic Versus Conventional Whole Rye and Wheat Flours for Making Sourdough Bread: Safety, Nutritional, and Sensory Implications

**DOI:** 10.3389/fmicb.2021.674413

**Published:** 2021-07-12

**Authors:** Erica Pontonio, Kashika Arora, Cinzia Dingeo, Ilaria Carafa, Giuseppe Celano, Valentina Scarpino, Bernard Genot, Marco Gobbetti, Raffaella Di Cagno

**Affiliations:** ^1^Department of Soil, Plant and Food Science, University of Bari Aldo Moro, Bari, Italy; ^2^Faculty of Science and Technology, Free University of Bozen-Bolzano, Bolzano, Italy; ^3^Department of Agricultural, Forest and Food Sciences, University of Turin, Grugliasco, Italy; ^4^Puratos srl, Groot – Bijgaarden, Belgium

**Keywords:** organic, wheat, rye, sourdough, lactic acid bacteria, bread, mycotoxin, nutritional profile

## Abstract

Organic farming is gaining a broad recognition as sustainable system, and consumer demand for organic products has increased dramatically in the recent past. Whether organic agriculture delivers overall advantages over conventional agriculture is, however, contentious. Here, the safety, nutritional, and sensory implications of using commercial organic rye, soft, and durum wheat flours rather than conventional-made sourdough bread have been investigated. Culture-dependent and culture-independent approaches were used to explore the microbial architecture of flours and to study their dynamics during sourdough propagation. Besides biochemical features, the main nutritional (amino acid content, asparagine level, and antioxidant activity) characteristics of sourdoughs were investigated, and their effect on the structural, nutritional, and sensory profiles of breads assessed. Overall, the organic farming system led to flours characterized by lower content of asparagine and cell density of Enterobacteriaceae while showing higher concentration of total free amino acids. Differences of the flours mirrored those of sourdoughs and breads. The use of sourdough fermentation guaranteed a further improvement of the flour characteristics; however, a microbial and sensory profile simplification as well as a slight decrease of the biochemical parameters was observed between breads with sourdough after one-cycle fermentation and 10 days of propagation.

## Introduction

Developing sustainable food systems considered as a high priority by intergovernmental organizations has contributed to the increase of the production and consumption of organic food worldwide (Eu Agricultural Markets Briefs, 2019; Popa et al., 2019). Due to the green concept and constraints of the organic farming practices, consumers perceived organic foods as healthier as and more eco-sustainable than convenional ones (Liang and Lim, 2020). In front of two products on the market shelf, one conventional and the other organic, the consumer is led to buy the latter. The consumers’ preference for organic foods is, in fact, mainly associated with the concept related to wellbeing, animal welfare, sustainability, and environmental protection (Kesse-Guyot et al., 2018). This might be due to the higher content of bioactive compounds and lower content of unhealthy compounds such as synthetic fertilizers and pesticides in organic foods compared with conventional agricultural products (Vinković Vrček et al., 2014).

Cereals and their based products are among the vital staple foods (Brera et al., 2013), and bread is a traditional food with an average of consumption among European people of 59kg of bread per year (Pontonio et al., 2018). Despite a quite simple formulation, the Italian bread-making tradition displays an array of high-quality artisanal bread types (De Boni et al., 2019), including organic, whole meal, and durum wheat breads. Consumers are increasingly oriented to prefer healthy and highly nutritious bread, such sourdough-based, fat-free, low salt, whole grain, and local bread (De Boni et al., 2019). The label organic represents another healthy attribute of bread recognized by consumers. However, following the recent pandemic issue, more consumers in Italy are buying flours to make bread at home, to which they attribute even more healthy value (De Boni et al., 2019; Coldiretti, 2020).

On the other side, agriculture is one of Europe’s biggest emitters of greenhouse gases (GHGs), accounting for around 10% of emissions across the European Union (Eurostat, 2017), and among all, crop production-related activities have a significant role, where cereals are the most grown groups of crops (circa 310 million tonnes in 2017) (Moudrý et al., 2018). In Italy, GHG emissions connected to the national agro-food production account for approximately 19% of the total national GHG emission. For bread production, the most burdensome phase is cultivation, mainly due to the use of pesticides and fertilizers and the agricultural activities performed (Notarnicola et al., 2017; Ingrao et al., 2018). However, on average, a yield reduction of 20–40% is reported for arable crops inorganic systems compared with conventional systems, with differences that strongly depend on site and system characteristics (Boone et al., 2019). With the aim of reducing emission by 30% by 2030, organic production represents one of the options available to move in the direction of more sustainable farming systems (Boone et al., 2019). Indeed, the evaluation of the GHG of the oat, rye, wheat, and spelt highlighted that the organic farming system contributes to lower GHG emissions than does the conventional one (Moudrý et al., 2018). When ascertained, the farming system may also affect the main features of the cereals and their products; indeed, higher content of phenolic compounds, vitamins, and minerals (Popa et al., 2019); protein digestibility (Yu et al., 2018); and microbial richness and diversity (Rizzello et al., 2015) were found in organic flours as compared with conventional ones. However, the emerging problems associated with cereals in terms of mycotoxin outbreaks and pathogens in both the cereals and their products are gaining significant attention (Hussain et al., 2019).

Here, we aim to provide an answer to consumers, millers, and bakers to reveal what is behind the organic label of commercial flours and how it can affect the safety, nutritional, and sensory features of traditional sourdough bread. The comparison of the commercially available *Triticum turgidum* spp. *durum* (durum wheat), *Triticum aestivum* (soft wheat), and *Secale cereale* (rye) flours, sourdough, and breads was addressed to investigate the microbial diversity; robustness of selected starters; presence of photogenes, mycotoxin, and acrylamide precursors; antioxidant potential; and *in vitro* protein digestibility (IVPD).

## Materials and Methods

### Flours

#### Gross Chemical Composition

Commercially available conventional and certified organic whole rye (*S. cereale*, cR and oR), soft (*T. aestivum*, cSW and oSW), and durum (*Triticum durum*, cDW and oDW) wheat flours were used in this study. Rye and soft wheat flours were purchased from local South Tyrol millers, while durum wheat flour was from a local South of Italy miller. The gross chemical compositions, as reported on the labels, for cR, cSW, cDW, oR, oSW, and oDW were, respectively, as follows: moisture, 7.8, 8.9, 13.7, 8.3, 8.9, and 12.6%; protein (N X 5.7), 8.2, 13.0, 12.5, 9.8, 13.2, and 12.5%, of dry matter (d.m.); total carbohydrates, 75.9, 72.0, 71.5, 55.1, 71.9, and 71.5% of d.m.; dietary fiber, 11.7, 11.0, 9.8, 19.7, 10.7, and 9.8% of d.m.; and fat 2.0, 2.5, 1.7, 2.1, 2.5, and 1.7% of d.m.

#### Multi-Mycotoxin Liquid Chromatography With Tandem Mass Spectrometry Analysis

Mycotoxin analysis was performed by a multi-mycotoxin liquid chromatography with tandem mass spectrometry (LC-MS/MS) method as described by Scarpino et al. (2019). Briefly, 5 g of the conventional and organic rye, soft, and durum wheat flours was extracted with 20 ml of CH_3_CN/H_2_O/CH_3_COOH (79/20/1, v/v/v), according to the dilute-and-shoot method reported by Scarpino et al. (2019), and 20 μl of the diluted filtered extracts was analyzed without any further pretreatment. LC-MS/MS analysis was carried out on a Varian 310 triple quadrupole (TQ) mass spectrometer (Varian, Milan, Italy), equipped with an electrospray ionization (ESI) source, a 212 LC pump, a ProStar 410 AutoSampler and dedicated software. LC separations were performed on a Gemini-NX C18 100 × 2.0 mm i.d., 3 μm particle size, 110 Å, equipped with a C18 4 × 2 mm security guard cartridge column (Phenomenex, Torrance, CA, United States), using water (eluent A) and methanol (eluent B), both acidified with 0.1% v/v CH_3_COOH, as eluents that were delivered at 200 μl/min. The chromatographic and MS conditions and the results pertaining to the linearity range, the limit of detection (LOD), the limit of quantification (LOQ), the apparent recovery RA (%), the matrix effects obtained through the evaluation of the signal suppression/enhancement SSE (%), and the recovery of the extraction RE (%) were described in detail by Scarpino et al. (2019). The results of the mycotoxin concentrations were corrected for the recovery rates and expressed as μg/kg on dry matter basis.

#### Microbiological Characteristics

Ten grams of each flour was suspended in 90 ml of sterile sodium chloride (0.9%, w/v) solution and homogenized in a Stomacher lab blender (Bagmixer^®^ 400, Model P) for 2 min at room temperature. Presumptive lactic acid bacteria were determined on De Man, Rogosa and Sharpe (MRS) (Oxoid, Basingstoke, Hampshire, United Kingdom) agar medium and modified MRS (maltose and fresh yeast extract were added at 1% and 5%, respectively, and the final pH was 5.6) (mMRS) supplemented with cycloheximide (0.1 g/L, w/v) at 30°C for 48 h under anaerobiosis. Molds were enumerated on Potato Dextrose Agar (PDA; Oxoid) at 25°C for 48 h. Cell density of yeasts was estimated on Sabouraud Dextrose Agar (SDA; Oxoid), supplemented with chloramphenicol (0.1 g/L, w/v) at 25°C for 48 h. Total Enterobacteriaceae were determined on Violet Red Bile Glucose Agar (VRBGA; Oxoid) at 37°C for 24 h, and total bacteria were determined on Plate Count Agar (PCA; Oxoid) at 30°C for 48 h. Total anaerobes and facultative anaerobes were determined on Thioglycollate agar (TG; Biolife, Milan, Italy) at 30°C for 72 h and on Tryptic Soy Broth (Biolife) at 24°C for 30 h, respectively. Dextrose casein-peptone agar (Merck KGaA, Darmstadt, Germany) was used for the enumeration of *Bacillus cereus*, *Bacillus subtilis*, *Bacillus coagulans*, and *Geobacillus stearothermophilus* according to manufacturer’s instructions. Indeed, mesophilic and thermophilic bacteria were incubated up to 72 h at 35°C and up to 48 h at 55°C–60°C, respectively.

### Investigation of the Lactic Acid Bacteria Biota

#### Isolation, Genotypic Characterization, and Identification

Ten grams of each flour was suspended in 90 ml of mMRS broth (Oxoid) containing 0.1% (w/v) of cycloheximide, used as the enrichment medium, and incubated at 30°C for 48 h under stirring conditions (100 rpm) (Chen et al., 2005). Serial dilutions were made and plated on mMRS agar (Oxoid) at 30°C for 48 h. At least 10 colonies of presumptive lactic acid bacteria were randomly selected from the plates containing the two highest sample dilutions. Gram-positive, catalase-negative, non-motile rod and coccus isolates were cultivated in mMRS broth at 30°C for 24 h and re-streaked onto the same agar medium. All isolates were preliminary evaluated for the ability to acidify the mMRS broth when incubated at 30°C for 24 h. Only those able to acidify the culture medium were considered for further genomic DNA extraction and biotyping.

Genomic DNA from presumptive lactic acid bacteria was extracted using a DNeasy blood and tissue kit (Qiagen, SA, Courtaboeuf, France) according to the manufacturer’s instructions. The biotyping of isolates was performed as reported by Di Cagno et al. (2014). RAPD-PCR profiles were acquired by the MCE-202 MultiNA microchip electrophoresis system (Shimadzu s.r.l., Milan, Italy), using the DNA-2500 reagent kit (100–2,500 bp) and the 2-log DNA ladder (0.1–10.0 kb) (Promega Srl, Padua, Italy) according to the manufacturer’s instructions. The similarity of the electrophoretic profiles was assessed by Dice coefficients of similarity and the unweighted-pair group method by using average linkages (UPGMA) algorithm. To identify presumptive lactic acid bacterial strains, two primer pairs, LacbF/LacbR and LpCoF/LpCoR (Invitrogen Life Technologies, Milan, Italy), were used for amplifying the 16S rRNA genes (De Angelis et al., 2006). Primers designed for the recA gene (Torriani et al., 2001) were also used to distinguish *Lactiplantibacillus plantarum*, *Lactiplantibacillus pentosus*, and *Lactiplantibacillus paraplantarum* species (formerly known as *Lactobacillus plantarum*, *Lactobacillus pentosus*, and *Lactobacillus paraplantarum*, respectively) (Zheng et al., 2020). PCR products were separated by electrophoresis carried out on an agarose gel at 1.5% (w/v) (GellyPhor; EuroClone), and amplicons were purified by the Nucleospin gel and PCR clean-up kit (Macherey–Nagel, Düren, Germany). In addition, amplicons were subjected to Sanger sequencing (Sanger et al., 1977). rRNA sequence alignments were carried out using the multiple-sequence alignment method (Edgar, 2010), and identification queries were fulfilled by a BLAST search (Altschul et al., 1997) in GenBank^1^.

Strains showing homology of at least 97% were considered to belong to the same species (Goebel and Stackebrandt, 1994). Cultures were maintained as stocks in 15% (v/v) glycerol at −80°C and routinely propagated at 30°C for 24 h in mMRS broth.

#### Selection of Starter for Sourdough Fermentation

Nine strains as representative of the whole flour lactic acid bacteria biota were cultivated into mMRS (Oxoid) broth at 30°C for 24 h. Cells were harvested by centrifugation (10,000 × *g*, 10 min, 4°C), washed twice in 50 mM of sterile potassium phosphate buffer (pH 7.0), re-suspended in tap water at the cell density of circa 9.0 log10 cfu/ml, and used as starters (cell density in the dough of 7.1 ± 0.3 log10 cfu/g) for conventional and organic flour fermentation. Dough yield (DY; dough weight × 100/flour weight) was 160 and 180 when wheat and rye flours were used, respectively. The mixing was done manually for 5 min, and fermentations were carried out in triplicate at 25°C for 24 h. Acidification and increase of cell density were used as screening criteria. The best-performing strains were selected and used as a mixed starter for sourdough fermentation of conventional and organic flours.

### Sourdough Fermentation

#### Preparation and Propagation

To compare sourdoughs within the same cereal (rye, soft, or durum wheat flours), the same binary starter composed of *L. plantarum* and *Pediococcus acidilactici* was used to ferment conventional and organic rye (oR4 and oSW5), soft (oSW3 and oSW5), and durum wheat (DW9 and oSW5) flours depending on the source of isolation of *L. plantarum* strains. While the strain of *L. plantarum* differed among cereals (oR4, oSW3, or DW9), the same *P. acidilactici* (oSW5) strain was used for all flours.

#### Sourdoughs

Dough preparation and bacterial inoculum were carried out as reported above. The same cell density (7.1 ± 0.3 log10 cfu/g) for all lactic acid bacteria strains was used among flours. To ensure the leavening power of the sourdough, a yeast strain of *Saccharomyces cerevisiae* N2, previously isolated and selected according to the leavening capacity and positive sensory dough profile, was inoculated at the cell density of 6.2 ± 0.3 log10 cfu/g of dough for all flours. Hence, the ratio between lactic acid bacteria and yeast (1:10) was used in each mixed starter. Daily, each sourdough was subjected to fermentation (propagation) at 25°C for 5 h (Ercolini et al., 2013). The only exception was the first fermentation, which lasted 24 h. After each daily fermentation, sourdoughs have quickly been cooled (circa 1 h) using ice-bath and stored at 2°C for circa 16 h. Sourdough propagation was done according to the back-slopping (refreshment) procedure, where the sourdough from day before was used as starter (25%, w/w, of inoculum) to ferment a new mixture of flours and water. With the aim of investigating the microbial dynamics during sourdough propagation, doughs prior (cR0, oR0, cDW0, oDW0, cSW0, and oSW0), after the first fermentation (cR1, oR1, cDW1, oDW1, cSW1, and oSW1), and after 1 (cR2, oR2, cDW2, oDW2, cSW2, and oSW2), 5 (cR5, oR5, cDW5, oDW5, cSW5, and oSW5), and 10 (cR10, oR10, cDW10, oDW10, cSW10, and oSW10) days of propagation were used for microbiological analysis. However, due to the use of t1 and t10 sourdoughs as leavening agent in breadmaking, doughs and sourdoughs at t0, t1, and t10 were used for biochemical and nutritional characterization. The only exception was the pH, which was daily measured aiming at monitoring the fermentation. Sourdoughs were cooled down to 4°C and analyzed within 2 h after collection. Preparation and propagation of each sourdough were carried out in triplicate.

#### Microbiological Characterization

Presumptive lactic acid bacteria and yeast were enumerated on doughs (t0) and sourdoughs (t1, t2, t5, and t10) as specified above. *Pseudomonas* and *Aeromonas* taxa were determined on GSP Agar (Oxoid) at 28°C for 48 h. *Staphylococcus* and Micrococcaceae were determined on Mannitol Salt Agar (MSA; Biolife, Milan, Italy) at 24°C for 48 h. Coliforms were determined on Chromocult^®^ Coliform Agar at 37°C for 37 h. *Escherichia coli* and *Enterobacter aerogenes* were determined on Eosin Methylne Agar Levine at 37°C for 24/48 h.

##### Robustness of starters

With the aim of evaluating the robustness of the mixed starters during the sourdough propagation, presumptive lactic acid bacteria were isolated from sourdoughs at t1, t2, t5, and t10. At least 15 colonies were randomly selected from mMRS (Oxoid) plates containing the three highest sample dilutions. Isolation and genotyping characterization were carried out as reported above. RAPD-PCR profiles were compared with those of the inoculated strains to assess the similarity.

##### Microbiota evolution through high-throughput sequencing

###### DNA extraction and PCR amplification

With the aim of investigating the autochthonous microbial architecture and dynamics during the sourdough propagation in the presence of added selected starter, high-throughput sequencing was carried out on doughs and sourdoughs.

Total DNA was extracted doughs prior (cR0, oR0, cDW0, oDW0, cSW0, and oSW0) and after the first fermentation (cR1, oR1, cDW1, oDW1, cSW1, and oSW1), and after 1 (cR2, oR2, cDW2, oDW2, cSW2, and oSW2), 5 (cR5, oR5, cDW5, oDW5, cSW5, and oSW5), and 10 (cR10, oR10, cDW10, oDW10, cSW10, and oSW10) days of propagation. Samples were processed according to the following procedure in order to reduce the contamination with chloroplast DNA (Minervini et al., 2010): 8 g of dough/sourdough sample was homogenized in 40 ml of sterile potassium phosphate-buffered saline (PBS; 50 mM, pH 7.0) followed by centrifugation at 200 × *g* for 5 min. The supernatant was then subjected to a second centrifugation step at 1,500 × *g* for 5 min at 4°C, and a further centrifugation at 14,000 × *g* for 15 min at 4°C. The pellet obtained was subjected to total DNA extraction by FastDNA Soil Kit (FastDNA Spin Kit for Soil; MP Biomedicals, Milan Italy). Quantification of total DNA was performed with Qubit^TM^ dsDNA HS Assay Kit (Thermo Fisher Scientific, Milan, Italy). Three independent replicates of each sample were used for DNA extraction and amplicon generation.

*Preparation of the MiSeq library*. Three DNA samples corresponding to the three biological replicates for each dough and sourdough were pooled and used for 16S and internal transcribed spacer (ITS)-based bacterial and fungal diversity analysis, respectively. Primers targeting the 16S rRNA variable regions V3–V4 (*E. coli* position 341–805, forward 341F: CCTACGGGNGGCWGCAG and reverse 806R: GACTACNVGGGTWTCTAATCC; Baker et al., 2003; Cleasson et al., 2010) of the 16S rRNA gene were used for bacteria, while primers (forward ITS1: CTTGGTCATTTAGAGGAAGTAA and reverse ITS2: CTGCGTTCTTCATCGATGC) targeting the ITS1 region between 18S and 5.8S rRNA genes were used for fungi (White et al., 1990; Gardens and Bruns, 1993).

Unique barcodes were attached to the forward primer for facilitating the differentiation of samples. Amplicons were cleaned using the Agencourt AMPure kit (Beckman Coulter, Brea, CA, United States) according to the manufacturer’s instructions, to prevent preferential sequencing of smallest amplicons, and DNA was quantified using the QuantiT PicoGreen dsDNA kit (Invitrogen). Amplicons were mixed and combined in equimolar ratios, and the quality and purity of the library were evaluated with the High Sensitivity DNA Kit (Agilent, Palo Alto, CA, United States) by the Bioanalyzer 2100 (Agilent). Library preparation and paired-end sequencing were carried out at the Genomic Platform—Fondazione Edmund Mach (San Michele a/Adige, Trento, Italy) using the Illumina MiSeq system (Illumina, San Diego, CA, United States) according to standard laboratory procedures.

*Illumina data analysis and sequences identification by QIIME2*. Raw paired-end FASTQ files were demultiplexed using idemp^2^ and imported into Quantitative Insights Into Microbial Ecology (Qiime2, version 2018.2). Sequences were quality filtered, trimmed, de-noised, and merged using DADA2 (Callahan et al., 2016). Chimeric sequences were identified and removed via the consensus method in DADA2. Representative bacterial sequences were aligned with MAFFT and used for phylogenetic reconstruction in FastTree using plugins alignment and phylogeny (Price et al., 2010; Katoh and Standley, 2013). Alpha- and beta-diversity metrics were calculated using the core-diversity plugin within QIIME2 and emperor (Vázquez-Baeza et al., 2013). Beta diversities were calculated using the Bray–Curtis distance matrix in QIIME2. Beta-diversity distance matrix indicates differences in taxa composition among samples based on either presence–absence or quantitative species abundance data. Output matrix was ordinated using principal coordinate analysis (PCoA) and visualized using EMPeror (Vázquez-Baeza et al., 2013). Bacteria taxonomic and compositional analyses were carried out by using plugins feature-classifier^3^. A naive Bayes taxonomy classifier was trained on the Silva (Quast et al., 2013) r132 reference sequences (clustered at 99% similarity) using q2-feature-classifier’s fit-classifier-naive-bayes method, trimmed to the V3–V4 regions of 16S rDNA and applied to paired-end sequence reads to generate taxonomy tables. Fungi sequences were classified to the species level with a 97% or 99% threshold (based on which is more accurate for certain lineages of fungi) by using UNITE v.8.0 Dynamic Classifier (Unite Community, 2019). Data on bacterial and yeast communities were subjected to one-way ANOVA; pairwise comparison of treatment means was obtained by Tukey’s procedure at *p* < 0.05, using the statistical software R 3.6.1 (R Core Team, 2019).

*Nucleotide sequence accession number*. The sequences are available in the Sequence Read Archive of National Center for Biotechnology Information (NCBI) (accession number PRJNA632578).

#### Biochemical Characterization

The values of pH were determined by a pH meter (Model 507; Crison, Milan, Italy) with a food penetration probe. Total titratable acidity (TTA) was determined on 10 g of doughs (t0) and sourdoughs (t1 and t10) homogenized with 90 ml of distilled water and expressed as the amount (ml) of 0.1 M of NaOH to reach pH of 8.3.

Water/salt-soluble extracts (WSEs) from doughs and sourdoughs were prepared according to the method described by Weiss et al. (1993) and used for the determination of sugars (glucose and fructose), organic acids (lactic and acetic), and total free amino acids (TFAAs) and asparagine concentrations. Briefly, a sample containing 1 g of flour was suspended in 4 ml of 50 mM Tris–HCl (pH 8.8), incubated at 4°C for 1 h under stirring conditions (150 rpm) and centrifuged at 12,000 × *g* for 20 min. Sugars (glucose and fructose) were determined by high-performance LC (HPLC) using an ÄKTA Purifier system equipped with a Spherisorb column (Waters, Millford, MA, United States) and a PerkinElmer (Beaconsfield, United Kingdom) 200a refractive index detector operating at 32°C. Elution was at 32°C, with a flow rate of 1 ml/min, using acetonitrile 80% as mobile phase. Organic acids were determined by HPLC, using an ÄKTA Purifier system (GE Healthcare, Buckinghamshire, United Kingdom) equipped with an Aminex HPX-87H column (ion exclusion, Bio-Rad, Richmond, CA, United States), and an UV detector operating at 210 nm. Elution was at 60°C, with a flow rate of 0.6 ml/min, using H_2_SO_4_ of 10 mM as mobile phase (Rizzello et al., 2010). The quotient of fermentation (QF) was determined as the molar ratio between lactic and acetic acids.

#### Nutritional Characterization

For the analysis of the TFAA and asparagine, proteins and peptides in the samples were precipitated by addition of 5% (vol/vol) cold solid sulfosalicylic acid, holding the samples at 4°C for 1 h and centrifuging them at 15,000 × *g* for 15 min. The supernatant was filtered through a 0.22-μm-pore-size filter and diluted, when necessary, with lithium citrate (0.2 M, pH 2.2) loading buffer. TFAA and asparagine were analyzed by using a Biochrom 30+ series Automatic Amino Acid Analyzer (Biochrom Ltd., Cambridge Science Park, United Kingdom), equipped with a Li-cation-exchange column (4.6 × 200 mm internal diameter), using lithium citrate buffer eluents following the elution conditions recommended by the manufacturer. A mixture of amino acids at known concentrations (Sigma Chemical Co., Milan, Italy) was added with tryptophan, ornithine, and γ-aminobutyric acid (GABA) and used as standard. Amino acids were post-column derivatized with ninhydrin reagent and detected by absorbance at 440 nm (proline) or 570 nm (all the other amino acids).

The 2,2-diphenyl-1-picrylhydrazyl (DPPH) radical scavenging activity (RSA) was determined on the methanolic extract (ME) of doughs and sourdoughs according to the method reported by Pontonio et al. (2019). Briefly, 3 g of each sample was mixed with 30 ml of methanol (80%, vol/vol) to get ME. The mixture was purged with nitrogen stream for 30 min, under stirring condition, and centrifuged at 4,600 × *g* for 20 min. The supernatants (MEs) were transferred into test tubes, purged with nitrogen stream, and stored at ca. 4°C before analysis. The scavenging activity was expressed as follows: DPPH scavenging activity (%) = [(blank absorbance - sample absorbance)/blank absorbance] × 100. The value of absorbance was compared with 75 ppm butylated hydroxytoluene (BHT), which was used as the antioxidant reference. The reaction was monitored by reading the absorbance at 517 nm. Total phenols were determined on the ME of flours as described by Slinkard and Singleton (1977) and expressed as gallic acid equivalent.

### Breadmaking

#### Bread Preparation

Breads were produced, following the well-established typical Italian breadmaking method for sourdough bread, at the pilot scale plant the Department of Soil, Plant and Food Science (University of Bari, Italy). For both conventional and organic flours, two different experimental breads were prepared: one with the sourdoughs obtained after the first fermentation (B-cR1, B-oR1, B-cDW1, B-oDW1, B-cSW1, and B-oSW1) and one with the sourdough after 10 days of propagation (B-cR10, B-oR10, B-cDW10, B-oDW10, B-cSW10, and B-oSW10). Sourdoughs were prepared as reported above. For B-cSW, B-cDW, B-oSW, and B-oDW, the sourdoughs were used as leavening agent at 15% (w/w), mixed with the corresponding flours and water, incubated at 30°C for 3 h (step II), and baked at 220°C for 15 min. For B-cR and B-oR, sourdoughs were used at 40% (w/w), subjected to two consecutive incubations of 1 h at 30°C and 37°C, respectively, and baked at 240°C for 10 min and 220°C for 35 min. The level of sourdough fortifications as well as the time and temperature of the baking phase were optimized at laboratory level according to the best final volume of dough and bread and the number and homogeneity of the gas cells (data not shown). To exclude the effect of the baking temperature on the mycotoxin concentration, a bread made without the sourdough fortification was made and used as control (B-CT) only for mycotoxin determination.

#### Biochemical and Nutritional Characterization

The values of pH and TTA, concentration of organic acids, and TFAA content were determined as previously reported. The IVPD of breads was determined according to the method described by Rizzello et al. (2014). Samples were subjected to a sequential enzyme treatment mimicking the *in vivo* digestion in the gastrointestinal tract, and IVPD was expressed as the percentage of the total protein, which was solubilized after enzyme hydrolysis. IVPD was expressed as the percentage of the total protein solubilized after a sequential enzymatic treatment mimicking the *in vivo* digestion enzymatic hydrolysis. The protein concentration was determined following the Bradford method (Bradford, 1976). The level of contamination of mycotoxins in breads (B-cDW1, B-cDW10, B-cR1, and B-cR10) was analyzed as reported above.

#### Textural and Structural Characterization

The Texture Profile Analysis (TPA) of bread was carried out with a Universal Testing machine (model 3344; Instron, Norwood, MA, United States), equipped with 3.6-cm-diameter cylindrical probe and 1,000-N load cell. The chromaticity coordinates of the bread crust (obtained by a Minolta CR-10 camera) were also reported in the form of a color difference, dE^∗^ab, as follows:

dE*=ab(dL*)2+(da*)2+(db*)2

where dL^∗^, da^∗^, and db^∗^ are the differences for *L*, *a*, and *b*-values, respectively, between sample and reference (a white ceramic plate having L^∗^ = 67.04, a^∗^ = 2.44, and b^∗^ = 18.28).

The alveolus percentage of the bread crumb (ratio of the gas cell area on the total area of the bread slice) was evaluated after 24 h of storage using the image analysis technology. The UTHSCSA ImageTool software was used, as previously described by Rizzello et al. (2012). The specific volume of breads was measured using the rapeseed displacement method. In detail, bread loaf was weighed and placed into a 2-L beaker. After having completely covered bread by pouring rapeseeds in the beaker, the volume was measured (cm^3^). Then, bread was removed from the beaker, and the volume of rapeseeds alone was measured. Specific volume (cm^3^/g) was calculated as the difference between the two measured volumes, divided by bread weight (AACC Method 10-05.01 Guidelines for Measurement of Volume by Rapeseed Displacement) (AACC, 2000).

#### Volatile Organic Compound Profile of Breads

Gas chromatography/MS (GC/MS) analysis of volatile organic compounds (VOCs) of breads was carried out using the headspace solid-phase microextraction (HP-SPME) sampling technique. According to Pico et al. (2018), 0.750 g of crushed bread (crumb or crust) sample was placed into 20-ml glass vials and added with 10 μl of 4-methyl-2-pentanol (final concentration of 33 mg/L), as the internal standard. A PAL COMBI-xt autosampler (CTC combiPAL; CTC Analytics AG, Zwingen, Switzerland) was used to standardize the extraction procedure. The bread samples were equilibrated to 60°C for 10 min. For HS-SPME, a divinylbenzene/carboxen/polydimethylsiloxane (DVB/CARB/PDMS) (Supelco, Bellefonte, PA, United States) fiber was exposed to the sample headspace for 50 min. The injection was made under splitless mode into the port at 230°C, equipped with a Merlino sealed. A Clarus 680 (PerkinElmer, Beaconsfield, United Kingdom) GC equipped with a Rtx-WAX column (30m × 0.25 mm i.d., 0.25 μm film thickness) (Restek Superchrom, Milan, Italy) was used to thermally desorb and to separate the headspace VOCs. The column temperature was set initially at 35°C for 8 min; then increased to 60°C at 4°C/min, to 160°C at 6°C/min, and finally to 200°C at 20°C/min; and held for 15 min (Montemurro et al., 2020). Helium was used as the carrier gas at flow rate of 1 ml/min. A single-quadrupole mass spectrometer Clarus SQ8MS (PerkinElmer) was used to detect the different compounds; the source and transfer line temperatures were 250°C and 230°C, respectively. The MS detector system operated in scan mode with mass-to-charge ratio interval 35 to 300 Da (Cavallo et al., 2017). Each chromatogram was analyzed for peak identification by comparing (i) retention time (RT) of detected compound with those of provided pure standard for HPLC (Sigma-Aldrich, St. Louis, MO, United States) and (ii) experimental mass spectra with those of the National Institute of Standards and Technology database (NIST/EPA/NIH Mass Spectral Library with Search Program, data version NIST 05, software version 2.0d). A peak area threshold of > 1,000,000 and a match criterion of > 85% was used for VOC identification followed by manual visual inspection of the fragment patterns when required. The volatile compounds were quantified by dividing the peak areas of the compounds of interest by the peak area of the IS and multiplying this ratio by the initial concentration of the IS (expressed as μg/g of 2-methyl-pentanol).

### Statistical Analysis

Three batches per each flour were used in this study. From each batch of flour, three different sourdoughs (total of nine sourdoughs per each flour) were prepared and propagated. Three analyses per each sourdough were performed (total of 27 analyses per each flour). Each sourdough was used to make two breads (total of 18 breads each flour), which were analyzed in triplicate (54 analyses per each flour). Data were subjected to one-way ANOVA; pairwise comparison of treatment means was achieved by Tukey’s procedure at p < 0.05, using the statistical software Statistica 12.5 (StatSoft Inc., Tulsa, OK, United States). Data of volatile organic were subjected to permutation analysis using PermutMatrix (Caraux and Pinloche, 2005). Cluster analysis based on the simple matching coefficient and unweighted pair group method using arithmetic average (UPGMA) was used for isolate biotyping. PCoA based on the Bray–Curtis distance analysis was used to analyze the 16S RNA gene and ITS sequences.

## Results

### Flour Characterization

#### Microbiological and Nutritional Properties

Table 1 summarizes the microbiological and nutritional properties of conventional and organic rye, soft, and durum wheat flours. The whole rye flours (both organic and conventional) had the highest loads for mesophilic aerobic bacteria (circa 5.8 log10 cfu/g) being 1 order of magnitude higher than other flours. No significant (*p* > 0.05) differences were found among wheat flours (Table 1). Molds were found at higher cell density in cR (3.5 ± 0.5 log10 cfu/g) compared with the other flours, for which a similar load was found (circa 2 log10 cfu/g). Except for oR (4.7 ± 0.3 log10 cfu/g), yeasts were not found in 10 g of any flour. Mesophilic presumptive lactic acid bacteria were not found in any of the investigated flour (Table 1). Enterobacteriaceae were found only in conventional flours ranging between 2.4 ± 0.4 (cDW) and 4.7 ± 0.3 log10 cfu/g (cR). None of the investigated *Bacillus* species were found in flours.

**TABLE 1:** Nutritional and microbiological characterization of conventional and organic rye, soft, and durum wheat used in this study.

	**cR**	**cSW**	**cDW**	**oR**	**oSW**	**oDW**
***Microbiological characteristics***						
Mesophilic aerobic bacteria (log10 cfu/g)	5.8 ± 0.3^a^	4.7 ± 0.4^b^	4.6 ± 0.3^c^	5.8 ± 0.2^a^	4.7 ± 0.5^b^	4.6 ± 0.2^c^
Yeast (log10 cfu/g)	< 10 cfu/10 g	<10 cfu/10 g	< 10 cfu/10 g	4.7 ± 0.3^a^	< 10 cfu/10 g	<10 cfu/10 g
Molds (log10 cfu/g)	3.5 ± 0.3^a^	< 10 cfu/10 g	2.4 ± 0.3^b^	< 10 cfu/10 g	2.4 ± 0.5^b^	2.4 ± 0.2^b^
LAB (log10 cfu/g)	< 10 cfu/10 g	<10 cfu/10 g	< 10 cfu/10 g	<10 cfu/10 g	< 10 cfu/10 g	<10 cfu/10 g
Enterobacteriaceae (log10 cfu/g)	4.7 ± 0.3^a^	3.5 ± 0.6^b^	2.4 ± 0.4^c^	< 10 cfu/10 g	<10 cfu/10 g	< 10 cfu/10 g
Facultative anaerobes	+	+	−	+	+	−
Strictly anaerobes	−	−	+	−	−	+
***Nutritional characteristics***						
DON (μg/kg)	659 ± 27^a^	427 ± 8^b^	440 ± 18^b^	724 ± 20^a^	495 ± 12^b^	486 ± 8^b^
DON-3-G (μg/kg)	81 ± 8^a^	66 ± 3^*ab*^	54 ± 5^b^	74 ± 7^*ab*^	57 ± 1^*ab*^	59 ± 2^*ab*^
3-ADON (μg/kg)	63 ± 4^a^	30 ± 2^b^	23 ± 1^b^	72 ± 6^a^	31 ± 2^b^	26 ± 4^b^
15-ADON (μg/kg)	< LOQ^a^	<LOQ^a^	< LOQ^a^	<LOQ^a^	< LOQ^a^	<LOQ^a^
*DON TOT* (μg/kg)	*803*^a^	*523*^b^	*517*^b^	*870*^a^	*583*^b^	*571*^b^
ZEA (μg/kg)	< LOQ^a^	<LOQ^a^	< LOQ^a^	<LOQ^a^	< LOQ^a^	<LOQ^a^
ENN A (μg/kg)	< LOQ^a^	<LOQ^a^	< LOQ^a^	<LOQ^a^	< LOQ^a^	<LOQ^a^
ENN A_1_ (μg/kg)	< LOD^b^	<LOD^b^	50 ± 5^a^	< LOD^b^	<LOD^b^	< LOD^b^
ENN B (μg/kg)	104 ± 9^b^	< LOQ^c^	244 ± 9^a^	< LOQ^c^	<LOQ^c^	< LOQ^c^
ENN B_1_ (μg/kg)	26 ± 2^b^	< LOQ^c^	131 ± 10^a^	< LOQ^c^	<LOQ^c^	< LOQ^c^
*ENN TOT* (μg/kg)	*130*^b^	*< LOQ^c^*	*425*^a^	*< LOQ^c^*	*<LOQ^c^*	*< LOQ^c^*
MON (μg/kg)	25 ± 2^b^	< LOQ^c^	60 ± 6^a^	32 ± 2^b^	< LOQ^c^	<LOQ^c^

*Data of mycotoxin are reported on dry basis.*

*cR, conventional rye (*Secale cereale* L.) flour; cSW, conventional soft wheat (*Triticum aestivum*) flour; cDW, conventional durum wheat (*Triticum durum*) flour; oR, organic rye flour; cDW, oSW, organic soft wheat flour; oDW, organic durum wheat flour; LAB, presumptive lactic acid bacteria; DON, deoxynivalenol; DON-3-G, deoxynivalenol-3-glucoside; 3-ADON, 3-acetyldeoxynivalenol; 15-ADON, 15-acetyldeoxynivalenol; DON TOT, total deoxynivalenol (sum of DON, DON-3-G, 3-ADON, and 15-ADON); ZEA, zearalenone; ENN A, enniatin A; ENN A_1_, enniatin A_1_; ENN B, enniatin B; ENN B_1_, enniatin B_1_; ENN TOT, total enniatins (sum of ENN A, ENN A_1_, ENN B, and ENN B_1_). +/−, presence/absence; n.d., not detected; n.c., not calculable; LOD, limit of detection (DON = 4.2 μg/kg; ENN A_1_ = 0.6 μg/kg); LOQ, limit of quantification (15-ADON = 10 μg/kg; ZEA). Values in the same row with different superscript letters differ significantly (*p* <0.05).*

Facultative anaerobes were found in R and SW (both organic and conventional), while strictly anaerobes were found in DW (both conventional and organic) (Table 1).

The highest level of deoxynivalenol (DON), deoxynivalenol-3-glucoside (DON-3-G), and 3-acetyldeoxynivalenol (3-ADON) was detected only in cR, while the highest level of enniatins (ENN A, ENN A1, ENN B, and ENN B1) and moniliformin (MON) was found in cDW (Table 1). cR and cDW flours significantly differed from each other with regards to their total deoxynivalenol (DON TOT = sum of DON, DON-3-G, 3-ADON, and 15-ADON), total enniatins (ENN TOT = sum of ENN A, ENN A1, ENN B, and ENN B1), and moniliformin levels of contamination (Table 1). On the other hand, zearalenone (ZEA) and 15-acetyldeoxynivalenol (15-ADON) were always detected under the LOQ (Table 1).

#### Identification of Lactic Acid Bacteria and Strain Selection

Because lactic acid bacteria were not detectable in 10 g of flour, the enrichment on mMRS broth was carried out, and 50 isolates of Gram-positive, catalase-negative, non-motile cocci and rods able to acidify the medium were subjected to RAPD-PCR analysis (Supplementary Figure 1). The reproducibility of the RAPD fingerprints was assessed by comparing the PCR products obtained from three separate cultures of the same strain. Primers M13, P7, and P4 generated different patterns, which were used for cluster analysis. The diversity among the isolates ranged from circa 2.5% to 40% (Supplementary Figure 1). Clusters gathered isolates with a maximum level of diversity of 20%. Two isolates that did not group into clusters and nine isolates that were representatives of each cluster were identified by partial sequencing of the 16S rRNA as *L. plantarum* (five biotypes/clusters), *P. acidilactici* (1), *Enterococcus mundtii* (2), and *Enterococcus xiangfangensis* (3).

According to the data shown in the Supplementary Figure 1, different species and strains were found among cereals. In detail, conventional and organic soft wheat harbored different strains of *L. plantarum* (strain n. 4/5 and 3, respectively) and *E. mundtii* (s. 1 and s. 2), with the organic flour characterized by the presence of *P. acidilactici* (s. 1). *L. plantarum* strain (s. 1), different from strains found in soft wheat flours, was identified in both conventional and organic durum wheat flours. Moreover, *L. plantarum* was flanked by the *E. xiangfangensis* in both conventional (s. 2) and organic (s. 1) flours. Organic rye harbored only an *L. plantarum* (s. 2) strain different from all other identified, while no bacterial growth was found in rye flour even after the enrichment on mMRS.

Lactic acid bacteria strains were singly used to ferment conventional and organic flours at 25°C for 24 h. The most relevant decrease of pH as well as the highest lactic acid bacteria growth was observed when *L. plantarum* oR4, oSW3, and DW9 strains were used to ferment the corresponding flours (R, SW, and DW, respectively). Overall strains showed a growth of circa 2.0 log10 cfu/g. Among other species, *P. acidilactici* oSW5 showed the best growth and acidification performances in all flours (data not shown). *Enterococcus* strains showed the worst performances in terms of both acidification and growth capacity (data not shown). Based on the above results, *L. plantarum* oR4, oSW3, and DW9 strains were chosen as binary starters in association with *P. acidilactici* oSW5.

Binary starters were used to ferment conventional or organic rye (oR4 and oSW5), soft (oSW3 and oSW5), and durum wheat (DW9 and oSW5) flours depending on the source of isolation of *L. plantarum* strains.

### Sourdoughs

#### Microbiological Characterization: Sourdough Microbiota and Starters

Sourdough microbiota and starters were monitored through the propagation. Among all, *E. coli* and *E. aerogenes* were not detected in any dough prior to the first fermentation (t0). All other microbial groups were found with a cell density varying according to the dough, being overall lower than 3.0 log10 cfu/g (Table 2). Moreover, *Pseudomonas* and *Aeromonas* were not detected in oDW0, and staphylococci were not found in cR0 and oR0 (Table 2). After the first fermentation (t1), none of the microbial groups investigated (except for the starters) were detected in doughs, while as expected, lactic acid bacteria and yeasts (starters) were circa 2 log10 cfu/g higher than those of t0 (Table 2). After the first refreshment (t2), *Pseudomonas* and *Aeromonas* was grown up to circa 1 log10 cfu/g in oR2, while staphylococci were found in cDW2 and oDW2 (2.4 ± 0.3 log10 cfu/g). Coliforms were detected in all doughs except for cSW2 (Table 2). Among starters, if yeast density remained constant, a slight increase (circa 0.5 log10 cfu/g) of the lactic acid bacteria cell density was observed after the first refreshment (t2). Increases of the cell density of *Pseudomonas* and *Aeromonas* and coliforms were found after 5 days of propagation (t5), which significantly decreased at the end of propagation (t10). Overall, although with a low cell density and with different magnitude according to the flour, *Pseudomonas* and *Aeromonas* (oR10), staphylococci (cDW10), and coliforms (all except for cSW10) were found until the end of propagation (Table 2). The ratio between lactic acid bacteria and yeast was in the range 1:10 (cR10, oR10 and oDW10) to 1:100 (cSW10, cDW10 and oSW10).

**TABLE 2:** Microbial characterization of doughs prior (cR0, cSW0, cDW0, oR0, oSW0, and oDW0) and after 1 (cR1, cSW1, cDW1, oR1, oSW1, and oDW1), 2 (cR2, cSW2, cDW2, oR2, oSW2, and oDW2), 5 (cR5, cSW5, cDW5, oR5, oSW5, and oDW5), and 10 (cR10, cSW10, cDW10, oR10, oSW10, and oDW10) days of propagation.

	***Pseudomonas* and *Aeromonas* (Log10 cfu/g)**	***Escherichia coli* and *Enterobacter aerogenes* (Log10 cfu/g)**	**Staphylococci (Log10 cfu/g)**	**Coliforms (Log10 cfu/g)**	**LAB (Log10 cfu/g)**	**Yeast (Log10 cfu/g)**
**Conventional**						
cR0	2.5 ± 0.2^b^	< 10 ufc/10 g	<10 ufc/10 g	2.5 ± 0.4^b^	7.1 ± 0.1^b^	6.2 ± 0.1^b^
cR1	< 10 ufc/10 g	<10 ufc/10 g	< 10 ufc/10 g	< 10 ufc/10 g	9.4 ± 0.1^a^	7.9 ± 0.1^a^
cR2	< 10 ufc/1 g	<10 ufc/1 g	< 10 ufc/10 g	2.1 ± 0.1^b^	9.9 ± 0.1^a^	8.1 ± 0.2^a^
cR5	3.6 ± 0.5^a^	< 10 ufc/10 g	<10 ufc/10 g	1.2 ± 0.3^b^	9.9 ± 0.2^a^	8.1 ± 0.2^a^
cR10	< 10 ufc/10 g	<10 ufc/10 g	< 10 ufc/10 g	2.3 ± 0.3^b^	9.9 ± 0.2^a^	8.1 ± 0.2^a^
cSW0	2.3 ± 0.2^b^	< 10 ufc/10 g	1 ± 0.2^b^	1 ± 0.2^b^	7.2 ± 0.1^b^	6.2 ± 0.2^b^
cSW1	< 10 ufc/10 g	<10 ufc/10 g	< 10 ufc/10 g	< 10 ufc/10 g	9.6 ± 0.2^a^	8.1 ± 0.2^a^
cSW2	< 10 ufc/1 g	<10 ufc/10 g	< 10 ufc/10 g	< 10 ufc/10 g	9.9 ± 0.2^a^	7.8 ± 0.3^a^
cSW5	3.5 ± 0.6^a^	< 10 ufc/10 g	< 10 ufc/10 g	2.5 ± 0.2^b^	9.9 ± 0.2^a^	7.8 ± 0.3^a^
cSW10	< 10 ufc/10 g	< 10 ufc/10 g	< 10 ufc/10 g	< 10 ufc/10 g	9.9 ± 0.1^a^	7.8 ± 0.3^a^
cDW0	1.5 ± 0.3^c^	< 10 ufc/10 g	1 ± 0.2^b^	2.3 ± 0.4^b^	7.2 ± 0.2^b^	6.1 ± 0.1^b^
cDW1	< 10 ufc/10 g	< 10 ufc/10 g	< 10 ufc/10 g	< 10 ufc/10 g	9.5 ± 0.2^a^	7.8 ± 0.1^a^
cDW2	< 10 ufc/10 g	< 10 ufc/10 g	< 10 ufc/10 g	1.2 ± 0.4^c^	9.8 ± 0.1^a^	8.2 ± 0.3^a^
cDW5	< 10 ufc/10 g	< 10 ufc/10 g	1.3 ± 0.3^b^	< 10 ufc/10 g	9.8 ± 0.1^a^	8.2 ± 0.3^a^
cDW10	< 10 ufc/10 g	< 10 ufc/10 g	2.4 ± 0.3^a^	1.3 ± 0.4^c^	9.8 ± 0.2^a^	8.2 ± 0.3^a^
**Organic**						
oR0	2.4 ± 0.4^b^	< 10 ufc/10 g	< 10 ufc/10 g	2.4 ± 0.2^b^	7.1 ± 0.1^b^	6.1 ± 0.1^b^
oR1	< 10 ufc/10 g	< 10 ufc/10 g	< 10 ufc/10 g	< 10 ufc/10 g	9.4 ± 0.2^a^	8.1 ± 0.1^a^
oR2	1.0 ± 0.3^c^	< 10 ufc/1 g	< 10 ufc/10 g	2.3 ± 0.2^b^	9.9 ± 0.2^a^	7.9 ± 0.2^a^
oR5	3.6 ± 0.4^a^	< 10 ufc/10 g	< 10 ufc/10 g	3.5 ± 0.2^a^	9.9 ± 0.1^a^	7.9 ± 0.2^a^
oR10	1.0 ± 0.3^c^	< 10 ufc/10 g	< 10 ufc/10 g	2.3 ± 0.2^b^	9.9 ± 0.1^a^	7.9 ± 0.2^a^
oSW0	2.4 ± 0.4^a,b^	< 10 ufc/10 g	1.0 ± 0.1^b^	2.3 ± 0.2^a^	7.1 ± 0.3^b^	6.2 ± 0.3^b^
oSW1	< 10 ufc/10 g	< 10 ufc/10 g	< 10 ufc/10 g	< 10 ufc/10 g	9.5 ± 0.3^a^	8.1 ± 0.3^a^
oSW2	< 10 ufc/10 g	< 10 ufc/10 g	< 10 ufc/10 g	2.3 ± 0.2^b^	9.9 ± 0.2^a^	7.8 ± 0.3^a^
oSW5	2.4 ± 0.2^b^	< 10 ufc/10 g	< 10 ufc/10 g	2.5 ± 0.3^b^	9.9 ± 0.2^a^	7.8 ± 0.3^a^
oSW10	< 10 ufc/10 g	< 10 ufc/10 g	1.1 ± 0.3^b^	2.3 ± 0.2^b^	9.9 ± 0.2^a^	7.8 ± 0.3^a^
oDW0	< 10 ufc/10 g	< 10 ufc/10 g	1.3 ± 0.3^b^	2.4 ± 0.3^b^	7.1 ± 0.2^b^	6.1 ± 0.2^b^
oDW1	< 10 ufc/10 g	< 10 ufc/10 g	< 10 ufc/10 g	<10 ufc/10 g	9.6 ± 0.2^a^	7.9 ± 0.2^a^
oDW2	< 10 ufc/10 g	< 10 ufc/10 g	2.4 ± 0.3^a^	1.3 ± 0.2^b^	9.9 ± 0.1^a^	8.2 ± 0.3^a^
oDW5	< 10 ufc/10 g	< 10 ufc/10 g	1.3 ± 0.2^b^	2.3 ± 0.2^c^	9.9 ± 0.1^a^	8.2 ± 0.3^a^
oDW10	< 10 ufc/10 g	< 10 ufc/10 g	2.4 ± 0.3^a^	1.3 ± 0.2^c^	9.9 ± 0.2^a^	8.2 ± 0.3^a^

**Lactiplantibacillus plantarum* oR4, SW3, and DW9 strains were used as binary starters in association with *Pediococcus acidilactici* oSW5 to ferment conventional or organic rye (oR4 and oSW5), soft (oSW3 and oSW5), and durum wheat (DW9 and oSW5) flours depending on the source of isolation of *L. plantarum* strains. *Saccharomyces cerevisiae* N2 was used as yeast in all samples. Bacteria and yeast were inoculated at final cell density of circa 7 and 6 log10 cfu/g, respectively. The first fermentation (t1) was carried out at 25°C for 24 h and propagation, through back-slopping (25°C for 5 h), and lasted 10 days. The data are the means of three independent experiments ± standard deviations (*n* = 3).*

*cR, conventional rye (*Secale cereale* L.) flour; cSW, conventional soft wheat (*Triticum aestivum*) flour; cDW, conventional durum wheat (*Triticum durum*) flour; oR, organic rye flour; cDW, oSW, organic soft wheat flour; oDW, organic durum wheat flour; LAB, presumptive lactic acid bacteria. ^a–c^Values in the same column with different superscript letters differ significantly (*p* < 0.05).*

As regards the starter robustness, inoculated strains were isolated from each sourdough during the propagation (t1, t2, t5, and t10) and biotyping. Plates containing the three highest dilutions (7, 8, and 9 log10 cfu/g) were used for this purpose. After the first fermentation (t1), all sourdoughs harbored both *L. plantarum* (oR4, oSW3, and DW9) and *P. acidilactici* oSW5 (ratio 1:1). Regardless of the type of cereal and the farming system, the propagation (from t2) allowed the dominance of *L. plantarum* strains, which were the only strains found at the highest cell density (9 log10 cfu/g). However, *P. acidilactici* oSW5 was found at 8 log10 cfu/g in a ratio circa 20:80 with *L. plantarum* strains until t5. Moreover, from t5 to t10, *P. acidilactici* oSW5 was not found at 7 log10 cfu/g, and other biotypes appeared. Looking at the new strains that appeared during the propagation, when different cereals were compared, only 40% of the RAPD-PCR profiles overlapped, while the similarity among strains increased up to circa 60% when conventional and organic flours obtained from the same cereal were considered. Overall, all the new strains belonged to *P. acidilactici* and *L. plantarum* species. The RAPD-PCR profiles of the main representative strains isolated from sourdoughs are shown in Supplementary Figure 2.

#### MiSeq Illumina Data Analysis, and Alpha and Beta Diversities

Quality-trimmed (3,946,569 and 3,359,686) and total (3,370,804 and 2,773,472) sequences for 16S rRNA gene and ITS1 region explained the entire bacterial and fungi diversity of the doughs and sourdoughs (Supplementary Figure 3). The number of observed operational taxonomic unit (OTU), Chao1, and Shannon indices is reported in Supplementary Table 1.

Overall, the highest diversity indices were found for the doughs (t0), especially for those made with rye flours, regardless of the farming system. Bacterial diversity remained stable after the first fermentation (t1) and the first refreshment (t2) and then decreased (Supplementary Table 1). A similar trend was found for the yeast diversity except for a slight increase after the first fermentation. Microbial diversity was also evaluated using phylogeny-based beta-diversity measures, namely, the PCoA based on the Bray–Curtis distance matrix (Supplementary Figures 4A,B). PCoA for bacteria differentiated samples based on the fermentation and propagation time. Indeed, doughs and t1- and t2-sourdoughs were grouped on the right side of the PCoA, whereas t5- and t10-sourdoughs were separated on the left part of the plane (Supplementary Figure 4A). Similarly, the distribution of samples on the plane, based on yeast diversity, seems to be partially affected by the propagation time PCoA rather than type of cereal or farming system (Supplementary Figure 4B).

#### Structure and Changes of the Dough and Sourdough Microbiota

Bacterial sequences from 16S rRNA genes assigned to bacterial phyla and their relative abundances (%) varied slightly depending on the flour and days of propagations (Supplementary Figure 5A).

**FIGURE 1:**
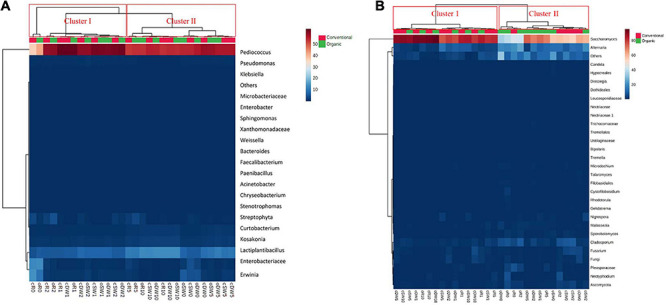
Pseudo heatmap depicting bacterial **(A)** and yeast **(B)** diversity. Relative abundance (%) in DNA samples directly from conventional (c) and organic (o) soft wheat (SW), durum wheat (DW), and rye (R) doughs prior (cR0, oR0, cDW0, oDW0, cSW0, and oSW0) and after (cR1, oR1, cDW1, oDW1, cSW1, and oSW1) the first fermentation and after 1 (cR2, oR2, cDW2, oDW2, cSW2, and oSW2), 5 (cR5, oR5, cDW5, oDW5, cSW5, and oSW5), and 10 (cR10, oR10, cDW10, oDW10, cSW10, and oSW10) days of propagation. For bacteria, only operational taxonomic units (OTUs) with an incidence above 0.01% in at least one sample are shown. For yeasts, the 30 dominant genera identified are shown. The color key de?nes the percentage of OTU in the samples. Further details are included in the section “MATERIALS AND METHODS.” Bacterial and yeast genera, and samples are sorted based on Euclidean distances.

The bacterial taxonomic composition at genus level is shown in Figure 1A. Based on the identified bacteria, doughs and sourdoughs were grouped in two clusters (I and II). Clustering was consistent with the PCoA (Supplementary Figure 4A). Cluster I included all doughs, t1- and t2-sourdoughs, whereas cluster II included t5- and t10-sourdoughs.

The bacterial taxonomic composition at species level is shown in Figure 1A. Based on the identified species, doughs and sourdoughs were grouped in two clusters (I and II). Clustering was consistent with the PCoA (Supplementary Figure 3A). Cluster I included all doughs, t1- and t2-sourdoughs, whereas cluster II included t5- and t10-sourdoughs. *Pediococcus* was the main genus found in conventional and organic DW (80.8%–77.7%, respectively), SW (76.0%–75.8%, respectively), and R (50.5%–57.4%, respectively). The species *Lactobacillus* was also detected in conventional and organic DW (11.6%–8.0%, respectively), SW (8.0%–9.5%, respectively), and R (6.1%–7.5%, respectively). However, pro?les of doughs made with conventional and organic rye flours differed for the higher abundance of Enterobacteriaceae (17.1%–18.0%) and *Erwinia* (19.1%–12.5%) compared with DW (0.9%–3.4% and 2.0%–6.4%, respectively) and SW (5.9%–2.4% and 5.8%–8.0%, respectively) doughs. The relative abundance of *Pediococcus* increased in all samples during propagations, showing a stabilization of relative abundance after 10 days (75.2%–76.1% in conventional and organic DW10, 76.4%–74.9% in conventional and organic SW10; and 74.4%–74.1% in conventional and organic R10). The species *Lactobacillus* remained almost stable in t1- and t2-sourdoughs and increased in t5- and t10-sourdoughs (17.8%–18.0% in conventional and organic DW10; 17.1%–17.9% in conventional and organic SW10; and 17.9%–17.4% in conventional and organic R10). Enterobacteriaceae and *Erwinia* decreased to less than 1% after 10 days’ propagation.

Sequences from ITS1 region assigned to yeast phyla and their relative abundances (%) varied slightly depending on the flours and days of propagations (Supplementary Figure 5B). All doughs were dominated by Ascomycota (from 63.4% to 82.9%), with low relative abundances of Basidiomycota, which varied depending on the flour and farming condition. In fact, the highest relative abundance was found for oR0 and cR0 (12.3% and 5.1%, respectively), followed by cSW0 and oSW0 (4.7% and 3.3%, respectively) and oDW0 and cDW0 (3.5% and 2.4%, respectively). Ascomycota dominated all t1-sourdough, whereas Basidiomycota decreased, reaching values between 0.4% and 1.5% and remained almost stable during propagation. Mature sourdoughs were dominated by Ascomycota (79.6%–95.4%), followed by others (2.2%–19.0%), Basidiomycota (0.8%–3.0%), and fungi (0.16%–1.5%). Yeast taxonomic composition at the genus level grouped doughs and sourdoughs into two clusters (I and II) (Figure 1B) consistently with the PCoA (Supplementary Figure 4B). Cluster I included t1-, t5-, and t10-sourdoughs, whereas cluster II included all doughs and almost t2-sourdoughs. *Saccharomyces* dominated doughs prior fermentation (32.8%–63.6%). Apart from the farming condition, the highest abundance was found in SW (56.8% and 56.2%, organic vs. conventional) and DW (62.3% and 35.3%, conventional vs. organic), followed by R (43.9% and 38.9%, conventional vs. organic). *Alternaria* was the second dominant genus found in DW (18.8%–1.0%), DW (13.6%–2.2%), and R (25.4%–2.2%). Pro?les of the cR0 and oR0 differed for the higher abundance of *Pleosporaceae*_other (6.8%–3.5%) compared with DW0 (0.08%–0.3%) and SW0 (0.1%–0%), and *Neotyphodium* (6.8%), which was only detected in dough made with oR. In addition, the profile of cSW and cDW was characterized by the higher abundance of *Fusarium* (4.4% and 5.0%) compared with their organic counterparts (0.09% and 0.9%).

After the first fermentation, the relative abundance of *Saccharomyces* increased, especially in conventional and organic R1 and oDW1 sourdoughs (over 50%), while *Alternaria*, *Cladosporium*, and Acomycota_other decreased. After the first refreshment, a shift of the trend was observed; indeed, *Saccharomyces* decreased, and *Alternaria*, *Cladosporium*, and Acomycota_other increased. The yeast profile furtherly changed in favor of *Saccharomyces*, which became the dominant genus, followed by *Alternaria*. Mature cDW10 (5.3%), cSW10 (1.0%), and cR10 (0.07%) sourdoughs contained a higher relative abundance of *Fusarium* as compared with their organic counterparts (0.1%, 0.3%, and 0.01%, respectively). Apart from the time of propagation, the abundance of *Fusarium* in conventional soft (4.4%–0.5%) and durum wheat (5%–0.2%), Fungi_other in conventional durum wheat (4.2%–0.1%), and *Nigrospora* in organic soft wheat (3.7%–0.1%) was significantly (*p* = 0.02, *p* = 0.01, *p* = 0.03, respectively) higher as compared with the other samples.

#### Biochemical and Nutritional Features

Biochemical and nutritional features of the doughs and sourdoughs during the propagation are summarized in Table 3. The pH-value in the dough prior the first fermentation (t0) was circa 6.0, being the highest for cR0 and oR0. TTA was the highest in cR0 and cDW0, which was also significantly higher than the corresponding organic doughs (oR10 and oDW10) (Table 3). Fructose was found at higher concentrations in organic wheat doughs (oSW0 and oDW0) as compared with the corresponding conventional ones (cSW0 and cDW0), while an opposite trend was found in rye doughs, where the concentration was higher in cR0 rather than in oR0 (Table 3). As regards glucose, significantly (p < 0.05) similar concentrations were found between organic wheat doughs (oSW0 and oDW0) and the corresponding conventional ones (cSW0 and cDW0), while oR0 contained lower concentration than cR0 (Table 3). While lactic acid was found in all doughs, being significantly (p < 0.05) higher in oR0 and oDW as compared with the corresponding conventional doughs, acetic acid was not detectable in any dough prior the fermentation.

**TABLE 3:** Biochemical and nutritional characteristics of doughs prior (cR0, cSW0, cDW0, oR0, oSW0, and oDW0) and after 1 (cR1, cSW1, cDW1, oR1, oSW1, and oDW1) and 10 (cR10, cSW10, cDW10, oR10, oSW10, and oDW10) days of propagation.

	**cR**	**cSW**	**cDW**	**oR**	**oSW**	**oDW**
**t0**						
**Biochemical properties**						
pH	6.15 ± 0.06^a^	5.91 ± 0.04^b^	5.88 ± 0.06^b^	6.12 ± 0.04^a^	5.85 ± 0.05^b^	5.93 ± 0.04^b^
TTA (ml of NaOH 0.1 M)	4.2 ± 0.2^b^	2.6 ± 0.2^cd^	4.6 ± 0.1^a^	1.8 ± 0.3^*e*^	2.8 ± 0.3^c^	2.4 ± 0.1^d^
Glucose (% w/w)	7.5 ± 0.4^a^	7.9 ± 0.3^a^	7.7 ± 0.5^a^	4.6 ± 0.2^b^	7.2 ± 0.5^a^	7.2 ± 0.4^a^
Fructose (% w/w)	13.5 ± 0.7^a^	4.7 ± 0.4^d^	7.5 ± 0.4^c^	10.2 ± 0.6^b^	10.3 ± 0.8^b^	12.0 ± 0.6^a^
Lactic acid (mmol/kg)	3.5 ± 0.4^c^	5.3 ± 0.3^c^	5.9 ± 0.6^b^	5.9 ± 0.2^b^	2.9 ± 0.4^d^	7.0 ± 0.3^a^
Acetic acid (mmol/kg)	n.d.	n.d.	n.d.	n.d.	n.d.	n.d.
QF	n.d.	n.d.	n.d.	n.d.	n.d.	n.d.
**Nutritional features**						
TFAA (mg/kg)	431 ± 12^*bc*^	471 ± 15^a^	394 ± 20^d^	478 ± 11^a^	410 ± 23^c^	441 ± 20^b^
Total phenols (mmol/kg)	1.41 ± 0.13^b^	1.01 ± 0.08^c^	1.90 ± 0.07^a^	1.19 ± 0.10^c^	1.95 ± 0.09^a^	1.50 ± 0.09^b^
Radical scavenging activity (%)	19 ± 2^a^	17 ± 1^a^	15 ± 2^a^	16 ± 2^a^	17 ± 1^a^	12 ± 1^b^
**t1**						
**Biochemical properties**						
pH	3.82 ± 0.04^a^	3.84 ± 0.03^a^	3.71 ± 0.02^b^	3.83 ± 0.04^a^	3.77 ± 0.03^*ab*^	3.83 ± 0.02^a^
TTA (ml of NaOH 0.1 M)	15.4 ± 0.2^d^	15.8 ± 0.3^d^	19.2 ± 0.4^a^	15.3 ± 0.4^c^	18.6 ± 0.2^a^	16.2 ± 0.3^b^
Glucose (% w/w)	10.9 ± 0.8^a^	4.8 ± 0.3^d^	7.7 ± 0.3^b^	4.0 ± 0.6^d^	6.7 ± 0.4^c^	6.5 ± 0.2^c^
Fructose (% w/w)	11.5 ± 0.2^a^	3.4 ± 0.1^c^	3.6 ± 0.3^c^	8.8 ± 0.2^b^	8.4 ± 0.3^b^	8.7 ± 0.1^b^
Lactic acid (mmol/kg)	71.1 ± 0.2^c^	72.5 ± 0.4^b^	51.8 ± 0.5^*e*^	68.3 ± 0.5^d^	73.5 ± 0.3^a^	45.6 ± 0.4^*f*^
Acetic acid (mmol/kg)	13.6 ± 0.2^*e*^	21.1 ± 0.4^c^	23.0 ± 0.3^b^	16.4 ± 0.2^d^	29.3 ± 0.3^a^	22.9 ± 0.3^b^
QF	5.0	3.0	2.0	4.0	3.0	2.5
**Nutritional features**						
TFAA (mg/kg)	1005 ± 11^b^	829 ± 20^c^	712 ± 21^*e*^	1120 ± 12^a^	800 ± 14^c^	862 ± 10^b^
Total phenols (mmol/kg)	1.74 ± 0.12^*e*^	3.07 ± 0.16^b^	2.65 ± 0.14^c^	2.36 ± 0.11^d^	3.25 ± 0.18^b^	3.63 ± 0.15^a^
RSA (%)	81 ± 3^a^	49 ± 2^c^	52 ± 2^c^	64 ± 2^b^	41 ± 1^d^	66 ± 3^b^
**t10**						
**Biochemical properties**						
pH	4.45 ± 0.03^c^	4.17 ± 0.02^d^	4.82 ± 0.02^a^	4.55 ± 0.03^b^	4.42 ± 0.02^c^	4.36 ± 0.04^c^
TTA (ml of NaOH 0.1 M)	11.2 ± 0.2^c^	12.2 ± 0.3^b^	11.4 ± 0.3^c^	11.6 ± 0.2^c^	10.2 ± 0.1^d^	13.8 ± 0.3^a^
Glucose (% w/w)	5.3 ± 0.2^a^	1.9 ± 0.3^*c,d*^	1.6 ± 0.2^d^	2.2 ± 0.1^c^	2.9 ± 0.3^b^	2.6 ± 0.2^b^
Fructose (% w/w)	n.d.	n.d.	n.d.	n.d.	n.d.	n.d.
Lactic acid (mmol/kg)	55.9 ± 0.3^a^	52.0 ± 0.5^b^	42.1 ± 0.2^d^	45.2 ± 0.3^c^	45.6 ± 0.2^c^	40.1 ± 0.3^*e*^
Acetic acid (mmol/kg)	10.3 ± 0.2^*f*^	12.6 ± 0.3^d^	27.3 ± 0.3^a^	13.8 ± 0.2^c^	11.9 ± 0.5^*e*^	24.7 ± 0.4^b^
QF	5.4	4.0	2.0	3.0	4.0	2.0
**Nutritional features**						
TFAA (mg/kg)	763 ± 10^d^	668 ± 13^*e*^	795 ± 11^*bc*^	899 ± 30^a^	805 ± 22^b^	864 ± 20^*ab*^
Total phenols (mmol/kg)	1.90 ± 0.11^*e*^	4.62 ± 0.31^a^	3.38 ± 0.17^b^	2.21 ± 0.09^d^	3.51 ± 0.23^b^	2.79 ± 0.31^c^
RSA (%)	70 ± 1^b^	62 ± 3^cd^	58 ± 2^d^	65 ± 3^c^	65 ± 2^c^	82 ± 4^a^

*The sourdoughs were used as leavening agent at 15% and 40% (w/w) in wheat and rye breads, respectively. *Lactiplantibacillus plantarum* oR4, SW3, and DW9 strains were used as binary starters in association with *Pediococcus acidilactici* oSW5 to ferment conventional or organic rye (oR4 and oSW5), soft (oSW3 and oSW5), and durum wheat (DW9 and oSW5) flours depending on the source of isolation of *L. plantarum* strains. *Saccharomyces cerevisiae* N2 was used as yeast in all samples. Bacteria and yeast were inoculated at final cell density of circa 7 and 6 log10 cfu/g, respectively. The first fermentation (t1) was carried out at 25°C for 24 h and propagation, through back-slopping (25°C for 5 h), and lasted 10 days. The data are the means of three independent experiments ± standard deviations (*n* = 3).*

*cR, conventional rye (*Secale cereale* L.) flour; cSW, conventional soft wheat (*Triticum aestivum*) flour; cDW, conventional durum wheat (*Triticum durum*) flour; oR, organic rye flour; cDW, oSW, organic soft wheat flour; oDW, organic durum wheat flour; QF, quotient of fermentation; TFAA, total free amino acids; RSA, radical scavenging activity; n.d., not detected.*

*^a–*f*^Values in the same row with different superscript letters differ significantly (*p* < 0.05).*

As regards nutritional features, doughs prior the fermentation were characterized by a concentration of TFFA of circa 400 mg/kg with a median value slightly higher for organic than conventional flours (441 mg/kg) (Table 3). A similar trend was found for total phenolic compounds, which ranged between 1.01 ± 0.1 and 1.95 ± 0.21 mmol/kg. RSA was less than 20% in all doughs. As expected, the first fermentation (t1), lasting 24 h, affected the biochemical and nutritional characteristics of sourdoughs. Significantly lower pH-values (3.71 to 3.84) were found without difference among samples. Accordingly, the concentration of organic acids as well as the TTA significantly (*p* < 0.05) increased. The highest TTA median value was found for organic sourdoughs (16.2 vs. 15.8). Although the acetic acid followed the same trend (22.1 vs. 21.1 mmol/kg), the median value of lactic acid was higher for conventional sourdoughs as compared with the organic ones (71.1 vs. 68.3 mmol/kg). Overall, the highest contents of acids were found in both conventional and organic soft wheat sourdoughs (cSW1 and oSW1). On the contrary, the concentration of fructose and glucose significantly (*p* < 0.05) decreased during the first 24 h of fermentation. A significantly different QF was found among sample according to the cereal used. However, the farming system did not affect the value. Overall, the highest and lowest values were found for rye and durum wheat, respectively, regardless of the farming system.

As regards the nutritional features, TFAA significantly increased (up to circa 130%), reaching the highest concentration in cR1 and oR1 (Table 3). The total phenols in the ME followed the same trend, being significantly higher in t1-sourdoughs as compared with the doughs prior the fermentation. The RSA reached values ranging from 41% to 81%, being the highest in cR1 and oR1 (Table 3).

The propagation led to changes of both biochemical and nutritional characteristics of the mature sourdoughs (t10). Overall, higher values of pH were mainly associated with the lower concentration of both lactic and acetic acids. However, QF did not change significantly between t1- and t10-sourdoughs. Although traces of glucose were still found in the t10-sourdoughs, fructose was not detected in any of these latter samples (Table 3). At the end of the propagation (t10), concentration of TFAA was higher in organic sourdoughs as compared with the conventional (Table 3). With the only exception of cR10, the propagation led to an increase of the RSA ranging from 62% to 82%.

**FIGURE 2:**
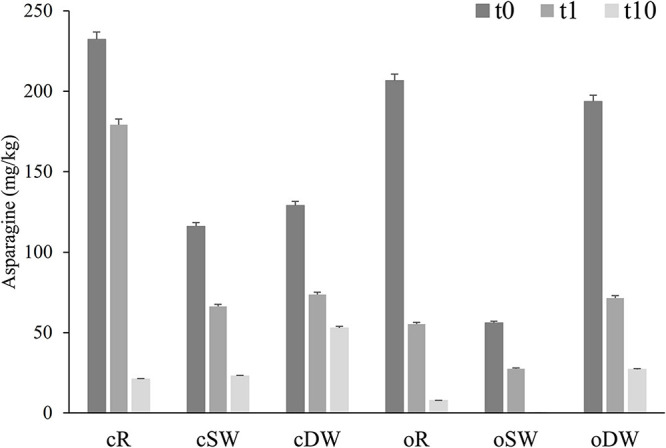
Concentration of asparagine found in doughs prior (cR0, oR0, cDW0, oDW0, cSW0, and oSW0) and after (cR1, oR1, cDW1, oDW1, cSW1, and oSW1) the first fermentation and after 10 days of propagation (cR10, oR10, cSW10, oSW10, cDW10, and oDW10). *Lactiplantibacillus plantarum* oR4, SW3, and DW9 strains were used as binary starters in association with *Pediococcus acidilactici* oSW5 to ferment conventional or organic rye (oR4 and oSW5), soft (oSW3 and oSW5), and durum wheat (DW9 and oSW5) flours depending on the source of isolation of *L. plantarum* strains. *Saccharomyces cerevisiae* N2 was used as yeast in all samples. Bacteria and yeast were inoculated at final cell density of circa 7 and 6 log10 cfu/g, respectively. The first fermentation (t1) was carried out at 25°C for 24 h and propagation, through back-slopping (25°C for 5 h), and lasted 10 days.

Due to the relevant involvement of the asparagine into the process of acrylamide formation, the content of the amino acid was monitored during the sourdough’s propagation (Figure 2). Overall, a significant (up to 26 times lower) decrease of the asparagine concentration was found during the propagation, which was almost higher in organic sourdoughs.

### Breads

#### Biochemical and Nutritional Features

Sourdoughs after the first fermentation (t1) and 10 days of propagation (t10) were used as leavening agents in breadmaking. The main biochemical (pH, TTA, lactic and acetic acids, and QF) and nutritional (TFAA and IVPD) characteristics were investigated. Overall, pH ranged between 5.17 ± 0.02 and 5.31 ± 0.03 when conventional and organic t1-SW and t1-DW were used. Lower values were found for rye breads (4.83 ± 0.02 and 4.81 ± 0.03), mainly due to the higher sourdough inoculum (40%, w/w). A similar trend was found for TTA values. Slightly but significantly (*p* < 0.05) higher values of pH were found when t10-sourdoughs were used in breadmaking. Indeed, pH ranged between 5.41 ± 0.03 and 5.48 ± 0.02 and 5.19 ± 0.01 and 5.22 ± 0.02 in B-c/oSW10, B-c/oDW10, and B-c/oR, respectively. Accordingly, a decrease of the TTA was found. Lactic and acetic acid concentration was found at levels of 21.8 ± 0.3 (B-oSW1) and 40.0 ± 0.1 (B-cR1) mmol/kg and 12.0 ± 0.2 (B-cDW1) and 18.2 ± 0.1 (B-cR1) mmol/kg, respectively. The concentration of lactic acid was found to be lower (circa 22%) only in B-cDW10 and B-oDW10, as compared with B-cDW1 and B-oDW1. Higher variations were found in terms of acetic acids, which decreased in four out of six samples and increased (circa 29%) in B-oR10 and B-cSW10. Bread coded as B-cSW and B-oSW showed the lowest QF (1.2), regardless of the type of sourdough used, while all breads showed QF of circa 2.

With regard to the nutritional features, the median concentration of TFAA in conventional and organic breads made with t1-sourdoughs was 330.6 and 303.7 mg/kg, respectively. When the t10-sourdoughs were used, the median values slightly increased in organic breads (circa 5% higher) and decreased in conventional breads (circa 14% lower). IVPD was significantly similar among samples, regardless of the cereal, farming system, and sourdough used, and being circa 92%.

**FIGURE 3:**
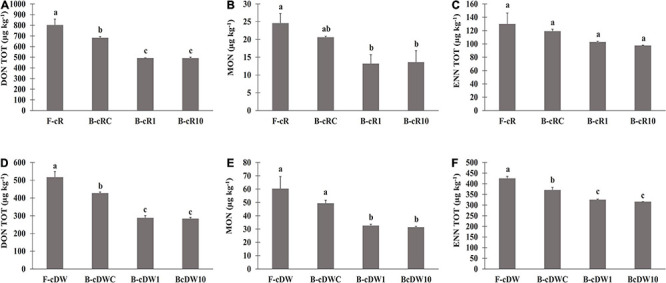
Mycotoxin levels in breads prepared with the sourdoughs obtained after the first fermentation (B-cR1 and B-cDW1) and those after 10 days of propagation (B-cR10 and B-cDW10). The sourdoughs were used as leavening agent at 15% and 40% (w/w) in wheat (DY, 160) and rye (DY, 180) breads, respectively. Lactiplantibacillus plantarum oR4 and DW9 strains were used as binary starters in association with Pediococcus acidilactici oSW5 to ferment conventional rye (oR4 and oSW5) and durum wheat (DW9 and oSW5) flours depending on the source of isolation of *L. plantarum* strains. Saccharomyces cerevisiae N2 was used as yeast in all samples. Bacteria and yeast were inoculated at final cell density of circa 7 and 6 log10 cfu/g, respectively. The first fermentation (t1) was carried out at 25°C for 24 h and propagation, through back-slopping (25°C for 5 h), and lasted 10 days. Panels **(A–C)** DON, MON and ENN levels in rye breads: Panels **(D–F)** DON, MON and ENN levels in durum wheat breads.

Due to the presence of the emerging mycotoxins only in cR and cDW (Table 1), the determination of these compounds was made only on breads B-cR1, B-cR10, B-cDW1, and B-cDW10. Breads not containing sourdough as leavening agent (B-cRC and B-cDWC) were used as the control (Table 4 and Figure 3).

**TABLE 4:** Mycotoxin levels in breads prepared with the sourdoughs obtained after the first fermentation (B-cR1 and B-cDW1) and those after 10 days of propagation (B-cR10 and B-cDW10).

	**B-cR1**	**B-cR10**	**B-cDW1**	**B-cDW10**
DON	410 ± 20^a^	406 ± 24^a^	244 ± 15^b^	243 ± 12^b^
DON-3-G	48 ± 0.5^*ab*^	52 ± 0.6^a^	34 ± 0.4^*ab*^	32 ± 0.3^b^
3-ADON	35 ± 0.4^a^	34 ± 0.4^a^	11 ± 0.2^b^	10 ± 0.2^b^
15-ADON	< LOQ^a^	< LOQ^a^	< LOQ^a^	< LOQ^a^
Total DON	493 ± 15^a^	492 ± 25^a^	288 ± 12^b^	284 ± 20^b^
ZEA	< LOQ^a^	< LOQ^a^	< LOQ^a^	< LOQ^a^
ENN A	< LOQ^a^	< LOQ^a^	< LOQ^a^	< LOQ^a^
ENN A1	< LOD^b^	< LOD^b^	33 ± 0.4^a^	30 ± 0.5^a^
ENN B	85 ± 0.7^b^	82 ± 0.6^b^	192 ± 20^a^	192 ± 14^a^
ENN B1	18 ± 0.3^c^	16 ± 0.2^c^	101 ± 11^a^	94 ± 0.7^b^
Total ENN	103 ± 10^c^	98 ± 0.7^d^	325 ± 30^a^	316 ± 27^b^
MON	13 ± 0.2^b^	14 ± 0.5^b^	33 ± 0.4^a^	31 ± 0.3^a^

*The sourdoughs were used as leavening agent at 15% and 40% (w/w) in wheat (DY, 160) and rye (DY, 180) breads, respectively. *Lactiplantibacillus plantarum* oR4 and DW9 strains were used as binary starters in association with *Pediococcus acidilactici* oSW5 to ferment conventional rye (oR4 and oSW5) and durum wheat (DW9 and oSW5) flours depending on the source of isolation of *L. plantarum* strains. *Saccharomyces cerevisiae* N2 was used as yeast in all samples. Bacteria and yeast were inoculated at final cell density of circa 7 and 6 log10 cfu/g, respectively. The first fermentation (t1) was carried out at 25°C for 24 h and propagation, through back-slopping (25°C for 5 h), and lasted 10 days. The data are the means of three independent experiments ± standard deviations (*n* = 3).*

*B-cR1, bread made with conventional rye (*Secale cereale*) flour using t1-sourdough; B-cR10, bread made with conventional rye flour using t10-sourdough; B-cDW1, bread made with conventional durum wheat (*Triticum durum*) flour using t1-sourdough; B-cDW10, bread made with conventional durum wheat flour using t10-sourdough; DON, deoxynivalenol; DON-3-G, deoxynivalenol-3-glucoside; 3-ADON, 3-acetyldeoxynivalenol; 15-ADON, 15-acetyldeoxynivalenol; DON TOT, total deoxynivalenol (sum of DON, DON-3-G, 3-ADON, and 15-ADON); ZEA, zearalenone; ENN A, enniatin A; ENN A1, enniatin A1; ENN B, enniatin B; ENN B1, enniatin B1; ENN TOT, total enniatins (sum of ENN A, ENN A1, ENN B and ENN B1). LOD, limit of detection (ENN A1 = 0.6 μg/kg); LOQ, limit of quantification (15-ADON = 10 μg/kg; ZEA = 1.2 μg/kg; ENN A = 1.6 μg/kg).*

*^a–d^Values in the same row with different superscript letters differ significantly (*p* < 0.05).*

Overall, similarly to what was recorded for flours, significantly higher contents of DON and of all its related forms (3-ADON and DON-3-G) were found in rye-based bread than durum wheat bread. On the other hand, an opposite trend was recorded for the ENN forms and for MON, which presented a significantly higher concentration in durum wheat bread than in rye ones (Table 4).

The level of DON was about 40% lower than flours, regardless of the cereal used. A similar behavior has also been recorded for the modified forms of DON, 3-ADON, and DON-3-G. Moreover, any statistical difference was found between t1- and t10-breads for all the detected DON forms. Instead, only 25% lower ENN was found in breads as compared with flours (Figure 3), with no significant differences between t1- and t10-breads, except for the ENN B1 in B-cDW and for the total enniatins both in B-cR and in B-cDW. Nevertheless, the highest reduction values from flour to bread were recorded for MON, reaching values of almost 50%, regardless of the cereal of the starting flour used (Figure 3). As far as DON TOT, MON, and ENN TOT contents were concerned, B-cR and B-cDW in comparison with the control breads (B-cRC and B-cDWC) recorded an average reduction of about −25% (DON TOT), −30% (MON), and −15% (ENN TOT), respectively, regardless the cereal and the time t1 and t10 of propagation. This decrease can thus be attributed to the presence of the sourdough. The remaining reduction recorded from flours to control breads, regardless of the cereal used, of about −15, −20, and −10% for DON TOT, MON, and ENN TOT, respectively, can instead be attributed to breadmaking (dilution factor due to the recipe) and baking processes.

#### Texture and Structure

Structural and textural characteristics of breads are summarized in Table 5. Breads made with t1-sourdoughs using DW were characterized by the highest specific volume, cohesiveness, gumminess, chewiness, and hardness, regardless of the farming system. However, B-oDW1 was characterized by lower values as compared with B-cDW1. When the t10-sourdoughs were used, the highest specific values were found for SW-breads; however, all the other above-mentioned characteristics were found at the highest values in B-oDW1 and c-oDW1 (Table 5). Opposite trends were found for these samples; indeed, cohesiveness, gumminess, chewiness, and hardness in B-oDW10 were higher than in B-oDW1; on the contrary, B-cDW1 was characterized by higher values than B-oDW10. According to the data collected, the color seems not to be affected by the type of sourdoughs and farming system; however, the cereal significantly affected the color of both crust and crumb. Overall, the lightness was higher in DW-containing breads as compared with the others. Moreover, rye-containing breads were characterized by the lowest values.

**TABLE 5:** Structural and textural characteristics of breads prepared with the sourdoughs obtained after the first fermentation (B-cR1, B-cSW1, B-cDW1, B-oR1, B-oSW1, and B-oDW1) and those after 10 days of propagation (B-cR10, B-cSW10, B-cDW10, B-oR10, B-oSW10, and B-oDW10).

	**B-cR**	**B-cSW**	**B-cDW**	**B-oR**	**B-oSW**	**B-oDW**						
**t1**												
Structural properties												
Specific volume (cm^3^/g)	1.46 ± 0.2^d^	1.60 ± 0.2^c^	1.95 ± 0.4^a^	1.69 ± 0.5^b^	1.55 ± 0.3^cd^	1.91 ± 0.3^a^						
Resilience	0.48 ± 0.3^*e*^	0.72 ± 0.3^c^	0.67 ± 0.4^d^	0.80 ± 0.4^b^	1.27 ± 0.3^a^	0.73 ± 0.4^c^						
Cohesiveness	0.12 ± 0.2^d^	0.33 ± 0.1^b^	0.40 ± 0.3^a^	0.30 ± 0.2^*bc*^	0.35 ± 0.4^a^	0.37 ± 0.2^a^						
Gumminess (N)	16.3 ± 0.2^*f*^	36.1 ± 1.4^*e*^	137.3 ± 10^a^	50.8 ± 2.3^c^	46.9 ± 0.7^d^	119.5 ± 0.3^b^						
Chewiness (g)	7.8 ± 0.1^*f*^	59.6 ± 2.^3c^	93.2 ± 1.7^a^	40.7 ± 1.5^d^	26 ± 0.8^*e*^	88.0 ± 1.7^b^						
Hardness (N)	134 ± 13^d^	129 ± 12^*de*^	336 ± 24^a^	164 ± 10^c^	108 ± 20^*ef*^	313 ± 20^*ab*^						

**Color**	***Crust***	***Crumb***	***Crust***	***Crumb***	***Crust***	***Crumb***	***Crust***	***Crumb***	***Crust***	***Crumb***	***Crust***	***Crumb***

L*	42.4 ± 1.5^d^	43.5 ± 1.7^*e*^	50.5 ± 1.8^*bc*^	53.7 ± 1.5^b^	53.9 ± 1.7^b^	58.3 ± 2.8^a^	43.5 ± 1.7^d^	46.9 ± 1.3^d^	52.7 ± 1.6^b^	48.9 ± 1.2^c^	59.5 ± 1.9^a^	60.4 ± 1.8^a^
a*	8.3 ± 0.4^a^	4.4 ± 0.3^b^	6.4 ± 0.3^c^	4.1 ± 0.3^c^	7.2 ± 0.4^b^	0.8 ± 0.3^d^	8.4 ± 0.5^a^	4.6 ± 0.3^*ab*^	6.7 ± 0.3^*bc*^	5.0 ± 0.3^a^	5.5 ± 0.4^d^	0.7 ± 0.4^d^
b*	26.7 ± 0.7^c^	20.7 ± 0.4^d^	25.8 ± 0.7^cd^	23.9 ± 0.5^b^	30.1 ± 0.8^a^	28.9 ± 0.9^a^	28.6 ± 0.2^b^	22.7 ± 0.5^c^	27.6 ± 0.4^c^	24.7 ± 0.5^b^	30.8 ± 0.6^a^	27.8 ± 0.4^a^
ΔE*	56.9 ± 0.8^a^	53.6 ± 0.6^a^	48.8 ± 0.8^b^	44.6 ± 1.7^c^	48.1 ± 0.6^b^	41.9 ± 0.8^*e*^	56.5 ± 0.5^a^	50.7 ± 0.7^b^	47.5 ± 0.7^*bc*^	49.5 ± 0.8^b^	42.8 ± 0.7^d^	42.8 ± 0.7^cd^
Crumb image analysis												
Black %	34.7 ± 1.8^*e*^	65.2 ± 0.5^c^	47.5 ± 1.5^d^	68.0 ± 1.5^b^	82.1 ± 1.5^a^	71.6 ± 0.9^b^						
White %	65.3 ± 1.3^a^	34.7 ± 0.6^c^	52.5 ± 1.7^b^	32.0 ± 1.3^c^	17.8 ± 0.4^*e*^	28.4 ± 1.4^cd^						
**t10**												
Structural properties												
Specific volume (cm^3^/g)	1.48 ± 0.3^b^	2.12 ± 0.5^a^	1.48 ± 0.2^b^	1.25 ± 0.2^c^	2.15 ± 0.3^a^	1.53 ± 0.3^b^						
Resilience	0.48 ± 0.3^c^	0.73 ± 0.3^b^	0.72 ± 0.6^b^	0.89 ± 0.3^a^	0.74 ± 0.6^b^	0.82 ± 0.4^a^						
Cohesiveness	0.12 ± 0.2^c^	0.35 ± 0.2^*ab*^	0.37 ± 0.4^*ab*^	0.27 ± 0.4^b^	0.34 ± 0.3^*ab*^	0.45 ± 0.3^a^						
Gumminess (N)	29.2 ± 1.2^*f*^	95.0 ± 1.2^d^	122.2 ± 12.6^c^	75.0 ± 1.5^*e*^	187.1 ± 20.3^a^	149.5 ± 10.3^b^						
Chewiness (g)	14.0 ± 0.9^*e*^	69.5 ± 1.8^d^	88.0 ± 1.8^c^	67.1 ± 1.8^d^	139.9 ± 10.3^a^	123.1 ± 15.3^*ab*^						
Hardness (N)	236 ± 20^cd^	261 ± 23^*bc*^	327 ± 35^b^	277 ± 30^b^	531 ± 32^a^	329 ± 25^b^						
L*	42.7 ± 1.7^d^	45.1 ± 1.5^*e*^	50.7 ± 1.8^*ab*^	59.1 ± 1.8^c^	51.7 ± 2.4^a^	65.8 ± 2.8^*ab*^	41.5 ± 1.5^*e*^	44.1 ± 1.4^*e*^	50.4 ± 1.6^*ab*^	57.3 ± 1.2^c^	46.9 ± 1.9^c^	66.7 ± 1.8^a^
a*	7.01 ± 0.9^*de*^	3.86 ± 1.4^*ab*^	7.9 ± 0.3^c^	1.6 ± 0.3^c^	8.7 ± 0.4^b^	0.73 ± 0.3	7.2 ± 1.3^d^	4.0 ± 0.6^a^	6.6 ± 1.2^*f*^	2.3 ± 1.2^d^	9.8 ± 0.4^a^	−1.56 ± 0.4
b*	26.7 ± 1.5^c^	21.6 ± 1.7^c^	28.9 ± 0.7^b^	20.1 ± 0.5^*e*^	32.1 ± 0.8^a^	25.7 ± 0.9^*ab*^	26.6 ± 0.7^cd^	20.8 ± 1.6^cd^	26.4 ± 0.4^cd^	20.4 ± 0.5^*e*^	32.1 ± 0.6^a^	26.4 ± 0.4^a^
ΔE*	56.6 ± 0.6^b^	52.2 ± 0.9^a^	50.2 ± 1.2^d^	38.1 ± 1.7^b^	50.6 ± 0.6^d^	33.8 ± 0.8^c^	57.6 ± 0.8^a^	52.9 ± 1.7^a^	49.3 ± 0.7^*de*^	39.9 ± 0.8^b^	55.3 ± 0.^7c^	33.3 ± 0.6^c^
Crumb image analysis												
Black %	77.05 ± 1.3^b^	64.70 ± 1.4^d^	52.63 ± 1.5^*e*^	83.25 ± 1.9^a^	73.87 ± 1.7^c^	65.81 ± 1.4^d^						
White %	22.95 ± 0.9^d^	35.30 ± 1.5^b^	47.37 ± 1.7^a^	16.75 ± 1.4^*e*^	26.13 ± 1.8^c^	34.19 ± 0.7^b^						

*The sourdoughs were used as leavening agent at 15% and 40% (w/w) in wheat (DY, 160) and rye (DY, 180) breads, respectively. *Lactiplantibacillus plantarum* oR4 and DW9 strains were used as binary starters in association with *Pediococcus acidilactici* oSW5 to ferment conventional rye (oR4 and oSW5) and durum wheat (DW9 and oSW5) flours depending on the source of isolation of *L. plantarum* strains. *Saccharomyces cerevisiae* N2 was used as yeast in all samples. Bacteria and yeast were inoculated at final cell density of circa 7 and 6 log10 cfu/g, respectively. The first fermentation (t1) was carried out at 25°C for 24 h and propagation, through back-slopping (25°C for 5 h), and lasted 10 days. The data are the means of three independent experiments ± standard deviations (*n* = 3).*

*^a–*e*^Values in the same row with different superscript letters differ significantly (*p* < 0.05).*

An image analysis procedure was performed for the characterization of the bread-crumb structure. The cell-total area corresponds to the black pixels, which was significantly affected by the cereal and farming system; indeed, values were higher on organic breads as compared with conventional breads. With regard to the cereal, the highest and lowest values were found in B-cSW1/B-oSW1 and B-cR1/B-oR1, respectively (Table 5). The use of t10-sourdoughs led to an increase of the cell-total area in conventional and organic rye and conventional durum wheat breads. Lower values were found in organic soft and durum wheat breads. No significant difference was found for conventional soft wheat bread (Table 5).

#### Volatile Organic Compounds

**FIGURE 4:**
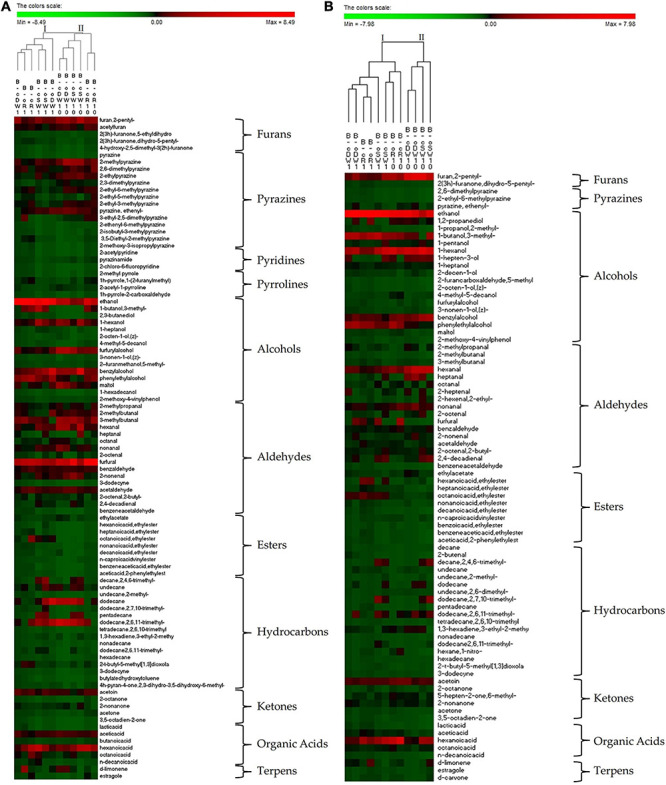
Permutation matrix of the relative abundances of the organic volatile compound identified in the crust **(A)** and crumb **(B)** of breads prepared with the sourdoughs obtained after the first fermentation (B-cR1, B-oR1, B-cDW1, B-oDW1, B-cSW1, and B-oSW1) and those after 10 days of propagation (B-cR10, B-oR10, B-cDW10, B-oDW10, B-cSW10, and B-oSW10). The sourdoughs were used as leavening agent at 15% and 40% (w/w) in wheat (DY, 160) and rye (DY, 180) breads, respectively. *Lactiplantibacillus plantarum* oR4 and DW9 strains were used as binary starters in association with *Pediococcus acidilactici* oSW5 to ferment conventional rye (oR4 and oSW5) and durum wheat (DW9 and oSW5) flours depending on the source of isolation of *L. plantarum* strains. *Saccharomyces cerevisiae* N2 was used as yeast in all samples. Bacteria and yeast were inoculated at final cell density of circa 7 and 6 log10 cfu/g, respectively. The first fermentation (t1) was carried out at 25°C for 24 h and propagation, through back-slopping (25°C for 5 h), and lasted 10 days.

The relative abundances of VOC in crust and crumb of breads have been investigated (Figures 4A,B). The permutation analysis of the VOC determined in the crust (Figure 4A) clearly showed that breads are divided into two clusters according to the type of sourdough used as leavening agent. Indeed, cluster I groups all breads made with t1-sourdoughs, while cluster II is composed of breads made with t10-sourdoughs (Figure 4A). Overall, breads made with organic flours contained significantly higher relative abundance (29.7 ± 0.9 μg/g) of VOC than their conventional counterparts (21.5 ± 0.4 μg/g), except for DW-breads for which the trend was opposite. As regards the DW-breads, B-cDW1 was characterized by significantly higher content of hydrocarbons and lower concentrations of furans (e.g., furan,2-pentyl-), alcohols (e.g., ethanol), aldehydes (e.g., 2-methylpropanal, 3-methylbutanal, heptanal, and furfural), ketones (e.g., acetoin and 2-nonanone), and organic acids, as compared with the B-oDW1 (Figure 4A). The effect of the farming system on the relative abundances of VOC was more evident when t10-sourdoughs were used (cluster II) for breadmaking. Indeed, except for DW, organic breads were characterized by higher relative abundance (28.1 ± 0.5 μg/g) as compared with their conventional counterparts (19.6 ± 0.8 μg/g) (Figure 4A). Moreover, R- and DW-breads contained the highest and lowest contents of VOC, respectively. When comparing the two clusters, an overall lower concentration of VOC was found in t10-sourdough breads as compared with t1-counterparts (21.9 ± 0.6 μg/g vs. 25.7 ± 0.4 μg/g). Significantly lower concentration of alcohols (e.g., ethanol 1-butanol, 3- methyl-, and phenylethyl alcohol), esters (e.g., octanoic acid and ethyl ester), ketones (e.g., acetoin), and organic acids were found in t10-sourdough breads, while higher concentrations of heterocyclic compounds (e.g., 2-methylpyrazine), aldehydes (e.g., furfural and 3-methylbutanal), and furans (e.g., furan,2-pentyl-) were found (Figure 4A).

According to the data of the bread crumb (Figure 4B), samples seem to be differentiated according to the sourdough used, except for R-breads, which are grouped in the same cluster, regardless of the leavening agent. Overall, regardless of the clusters, samples were characterized by similar relative abundances of VOC when the same cereal was used apart from the farming system. The only exception was the B-oR1, which contained a significantly higher concentration of volatile compounds as compared with B-cR1 (42.1 ± 0.5 μg/g vs. 29.1 ± 0.6 μg/g) mainly due to the higher relative abundance of alcohols (25.6 ± 0.7 μg/g vs. 15.5 ± 0.5 μg/g) (Figure 4B). When t1- and t10-sourdoughs were used, R- and SW-breads crumb were characterized by the highest relative abundance of VOC, respectively. Moreover, as already reported for the crust, the use of t10-sourdoughs led to lower relative abundance of VOC as compared with t1-counterparts (13.7 ± 0.8 μg/g vs. 25.3 ± 0.9 μg/g). Indeed, except for heterocyclic compounds and terpens, the median values of all other chemical classes of compound were significantly lower in t10- than t1-sourdoughs (Figure 4B).

## Discussion

Concerns about human health, and environmental and animal welfare as well as the will to support the local economy have been identified as the main purchase motives influencing consumer preference of organic foods (Hughner et al., 2007; De Boni et al., 2019). Differences in terms of chemical and nutritional features of the organic foods as compared with their conventional counterparts are still being debated. The farming system affects the total amount of plant nutrients, which results in a slight increase of nutritional value of organic crops compared with conventional ones (Popa et al., 2019).

Here, a comparison between commercial conventional and organic whole wheat and rye flours was made aiming at highlighting the differences in the flour–sourdough–bread axis. The microbial diversity of flours has been investigated. With regard to the culture-dependent methods, the farming system seems only to affect the cell density of Enterobacteriaceae, and although their presence represents one of the major concerns of the organic foods (Fernández-Fuentes et al., 2019), Enterobacteriaceae were detected only in conventional flours. On the contrary, the presence of other investigated microbial groups mainly depended on the cereal used. Cereals are commonly infected with various molds like *Aspergillus*, *Penicillium*, and *Fusarium*, which release secondary metabolites called mycotoxins (Vinković Vrček et al., 2014).

Although the content of mycotoxins is highly affected by different environmental and agronomical conditions and the comparison among samples could be made only when the same conditions were used, in this study, DON and associated forms were equally found in conventional and organic flours, while significantly higher contents of emerging mycotoxins were found in conventional rye and durum wheat. The lack of use of chemicals (including chemical fungicides) in organic production might favor the contamination with mycotoxin-producing molds (Lairon, 2010). However, this topic is still being debated with surveys indicating that organic production does not lead to overall noticeable differences in mycotoxin level (Vinković Vrček et al., 2014) and studies demonstrating that higher mycotoxin detection was found in organic compared with non-organic flours (Sacco et al., 2020).

Globally, cereal flours are used to prepare bread and pasta, with the former being the most consumed food worldwide (Sakandar et al., 2019), where sourdough bread is widely perceived as healthier, tastier, and more traditional than the baker’s yeast counterparts (De Boni et al., 2019). Apart from consumers’ choice, the sourdough potential as improver of the technological and nutritional attributes of conventional and non-conventional flours and to recycle agro-food by-products has largely been scientifically proven (Arora et al., 2021).

Traditional sourdough (Type I) is characterized by spontaneous fermentation, and the selection of sourdough microbiota occurs spontaneously during daily refreshment, also called back-slopping (Siepmann et al., 2018). Although sourdough may harbor complex microbial consortia (bacteria and yeast), most of its beneficial effects rely on the peculiar metabolic traits of few species/strains of naturally selected autochthonous lactic acid bacteria, mainly being heterofermentative (Gobbetti et al., 2016; Siepmann et al., 2018). The use of spontaneous sourdough requires hardwork, time-consuming processes, and skilled labor (Siepmann et al., 2018). Moreover, the stability of the sourdough microbiota during time is still being questioned, contributing to a certain variability of the products (Gobbetti et al., 2016). With the aim of standardizing the sourdough bread characteristics, managing the fermentation stage, and reducing the process time, industries are moving toward the use of Type II sourdough (Dolci and Cocolin, 2017). *Ad hoc*-selected lactic acid bacteria and, eventually yeast, are added to a mixture of flour and water (often under liquid conditions, DY > 200) to drive the fermentation (Siepmann et al., 2018). Here, autochthonous lactic acid bacteria were isolated from each flour and subjected to a selection process, mainly based on pro-technological parameter (fast growing and acidifying), to define a mixed starter to be used in Type II sourdough fermentation. Overall, numerous advantages of performing the starter selection among microorganisms isolated from the same environment in which they will be applied have been reported (De Vuyst and Neysens, 2005). Strains of *L. plantarum*, *P. acidilactici*, *E. mundtii*, and *E. xiangfangensis* were isolated. Although all the above genera have already been reported as part of the sourdough microbiota (De Vuyst et al., 2014), species of the genus *Enterococcus* were often identified at the early stages of the sourdough propagation, while *Lactiplantibacillus* and *Pediococcus* have been reported as sourdough-specific lactic acid bacteria (De Vuyst et al., 2014). Moreover, *L. plantarum* belongs to the well-adapted sourdough strains (De Vuyst et al., 2014; Gobbetti et al., 2016). In accordance with this differentiation, when singly used to ferment the flours, *L. plantarum* and *P. acidilactici* showed the best performances. *L. plantarum* strains have largely been reported (more than 70 scientific papers) as the most promising microbial starter for sourdough fermentation at laboratory (Arora et al., 2021) and industrial (Rizzello et al., 2016; Rizzello et al., 2020a, b) levels. Moreover, its use, together with *P. acidilactici*, has also been reported within the commercial starter (Brandt, 2014). Firm (DY 160) Type II sourdoughs were prepared by using binary starters consisting of a *L. plantarum* strain (SW3, DW9, or oR4) in association with *P. acidilactici* oSW5. Moreover, in order to ensure the leavening capacity of the sourdough, *S. cerevisiae* N2 was included (ratio 1:10, lactic acid bacteria:yeast) (Siepmann et al., 2018). With the aim of assessing the persistence of specific strains, the sourdoughs, initiated with the binary starter, were propagated with traditional back-slopping procedure (Minervini et al., 2012; Ercolini et al., 2013) leading to type 3 sourdoughs (based on the inoculum applied) (De Vuyst et al., 2017).

The dynamics of the taxonomic architecture of sourdoughs was monitored during propagation through culture-dependent and culture-independent approaches, and the main biochemical and nutritional features of the sourdoughs were investigated. With regard to the sequences for 16S rRNA gene and ITS1 region, the microbial diversity (bacteria and fungi) decreased during propagation, regardless of the type of cereal used and the farming system as previously reported for Type I sourdoughs (Ercolini et al., 2013; Rizzello et al., 2015). Moreover, doughs prior the fermentation and sourdoughs after the first fermentation (t1) and the first refreshment (t2) were grouped and separated by the sourdoughs after the fifth (t5) and 10th (10) refreshments. A clear effect of the flour and the stage of propagation on the doughs and sourdoughs microbiota has been highlighted. As expected, all sourdoughs were dominated by *L. plantarum* and *P. acidilactici* during the first fermentation and until 2 days of propagation. The presence of both starters is related to the decrease of the relative abundance of *Pantoea* after 1 day of fermentation and until 10 days of propagation. The increase of *L. plantarum* corresponded to a decrease of *P. acidilactici* after 5 and 10 days of propagation, suggesting the leading role of *L. plantarum* and the low competitiveness of *P. acidilactici* throughout propagations. Regarding the yeast community, *Saccharomyces* showed the highest relative abundance in all samples. The next-generation sequencing results were also confirmed by culture-dependent methods. Indeed, *L. plantarum* strains dominated throughout the propagation (cell density ≥ 8 log10 cfu/g), and *P. acidilactici* oSW5 progressively decreased, being not found at cell density ≥ 7 log10 cfu/g in any of the sourdoughs from the t6. Regarding the new strains, high similarity of RAPD-PCR profiles (up to circa 60%) was found when conventional and organic flours obtained from the same cereal were considered. The similarity decreased when different cereals were compared (40%). Overall, all the new strains belonged to *L. plantarum* and *P. acidilactici* species. The effect of the restricted number of isolated colonies should also be considered as responsible for the absence of the starter *P. acidilactici*. High persistence in back−slopping trials of inoculated *L. plantarum* strains has already been reported and mainly ascribed to its versatile metabolism and capacity of adaptation (Minervini et al., 2010; Dolci and Cocolin, 2017). The low competitiveness of the *Pediococcus* species in a mixed starter was recently demonstrated (Montemurro et al., 2020). Moreover, the occurrence of new biotypes during propagation shows that starters could be outcompeted by the flour and environmental microbial population (Meroth et al., 2003; Siragusa et al., 2009; Montemurro et al., 2020).

Overall, the fermentation with lactic acid bacteria led to biochemical and nutritional changes of all flours, with significant differences between t1- and t10-sourdoughs mainly due to the modification of the microbiota architecture that occurred during the propagation. The more acidic environment of sourdough might have better inhibited the growth of most of the microbial group investigated (Ercolini et al., 2013). The highest cell density of coliforms characterized overall organic sourdoughs. Because of the carcinogenic potential of acrylamide (Konings et al., 2020) formed by heating carbohydrate-rich food materials such as cereals (Stadler et al., 2002), the European Commission in 2017 has announced a regulation aiming to reduce the level of acrylamide (AA) in food products like cereals and potatoes (Commission Regulation (EU), 2017). Although several routes have been suggested for acrylamide formation in bread, the main precursors of acrylamide are free asparagine and reducing sugars (Mollakhalili-Meybodi et al., 2021), with the former being the main limiting precursor for AA in food (Mustafa et al., 2009). Among species, rye was characterized by the highest content of Asn as already reported by Stockmann et al. (2018). Moreover, except for durum wheat, organic flours contained lower concentrations than their conventional counterparts did. High fluctuations of Asn concentration from year to year have been found as results of several factors, i.e., the crop management, environmental conditions, and the cultivar considered (Stockmann et al., 2019). Bakery products contribute approximately 25% of the daily acrylamide exposure through the diet (Claus et al., 2008). Up to now, many technological measures have been considered for AA reduction in bakery products; however, the research of methods, which cause no alterations to the other properties of food products, is still challenging (Nasiri Esfahani et al., 2017). Here, the use of selected lactic acid bacteria and yeast led to the decrease on the concentration of both sugars (glucose and fructose) and Asn in all sourdoughs. Decrease in the ranges of 20%–70% and 60%–100% was found in t1- and t-10 sourdoughs, respectively. A similar trend was found for fructose and glucose, with the former not detected in any of the t10-sourdoughs and the latter decreased up to 80%. Recently, some sourdough lactic acid bacteria belonging to *Lactobacillus* and *Pediococcus* were potentially linked to acrylamide reduction in bakery products (Dastmalchi et al., 2016). Moreover, the role of baker’s yeast has also been highlighted, although the release of fructose might impair the overall effect (Konings et al., 2007). Nevertheless, investigation on bread dough prior baking and quantification of acrylamide in bread would give a more accurate information.

Sourdough breads were prepared, and results mirrored those found in the sourdoughs. Slight differences of the nutritional and biochemical characteristics were found according to the time of fermentation of the sourdough used as inoculum and its percentage. Indeed, lower pH and higher content of TFAA were detected in rye breads, where the highest percentage was used. Overall, sourdough breads showed similar characteristics to commercial samples (Pontonio et al., 2018) in terms of pH. The concentration of amino acids was > 300 mg/kg in all breads. These affect the taste of fermented foods and are important precursors for volatile flavor compounds, which are generated during baking (De Luca et al., 2021). The VOC profile of crust and crumb of breads revealed a high relative abundance of VOC associated with the flavor of baked goods (e.g., alcohols and aldehydes) (De Luca et al., 2021). The relative abundance of VOC in the crust of organic breads (31.3 ± 0.9 and 28.1 ± 0.2 μg/g in t1- and t10-breads, respectively) was higher than that of the conventional counterparts (23.3 ± 0.3 and 19.6 ± 0.6 μg/g in t1- and t10-breads, respectively), regardless of the propagation time, while similar relative abundances of VOC were found in crumb when the same cereal was considered.

Mineral and protein bioavailability and the presence of antinutritional factors have largely been reported as the main problems of whole flours (Poutanen et al., 2009). Fermentation with lactic acid bacteria has been shown to be an efficient pretreatment method for such matrices to overcome the above drawbacks (Poutanen et al., 2009; Gänzle, 2020). Here, apart from the cereal used, farming system, and time of propagation, breads were characterized by values of IVPD of circa 90% even higher than those of whole wheat, barley, and emmer bread fortified with fermented brans (Pontonio et al., 2020). Both endogenous enzymes and microbial activities might have to an intense proteolysis resulting in such high values of IVPD (Rizzello et al., 2019; Gänzle, 2020).

The use of sourdoughs led also to a decrease of the mycotoxin concentration in rye and durum wheat bread as compared with the control breads. The magnitude varied according to the cereal considered and the class of mycotoxin examined. Decreases from 8% to 18% of the mycotoxin concentration were found as results of the baking procedure. Overall, the effect of the baking step is particularly dependent on the heat stability of the mycotoxins (Schaarschmidt and Fauhl-Hassek, 2018); however, several studies found the baking time to be more important than the baking temperature (Schaarschmidt and Fauhl-Hassek, 2018). These findings might explain the higher decrease of mycotoxin concentration in rye control bread (compared with flour) than in durum wheat bread. The former, indeed, was baked longer (45 min) than the durum wheat counterpart (15 min). Although the data found in the literature on the effect of fermentation on mycotoxin levels are contradictory and require in-depth investigation, microbial-mediated degradation or transformation processes during fermentation cannot be excluded (Okeke et al., 2015; Schaarschmidt and Fauhl-Hassek, 2018; Adebo et al., 2019; Ademola et al., 2021). Besides, temperature/time regime of fermentation is a process parameter that can affect the residual concentration of mycotoxins (Banu et al., 2014). From 10% to 30% lower mycotoxin concentration was found in sourdough breads as compared with their respective controls. Moreover, the absorption of the mycotoxin on the cell wall of both lactic acid bacteria and yeast might not be excluded (Dalié et al., 2010).

## Conclusion

Under these study conditions, commercial organic flours showed a lower level of contamination in terms of cell density of Enterobacteriaceae, emerging mycotoxin, and asparagine concentration. On the contrary, higher contents of total free amino acids were found in organic flours. Differences of the flours reflected those of sourdoughs and breads. An improvement of the flour characteristics was guaranteed by sourdough fermentation. The comparison between sourdough obtained after one-cycle fermentation with selected starters and after 10 days of propagation highlighted the positive effect of the fermentation on the sourdough features; however, differences were found among sourdoughs. In turn, bread made with propagated sourdoughs showed a slight decrease of the biochemical parameters and simplification of the VOC profile, although the nutritional features were not significantly affected.

## Data Availability Statement

The datasets presented in this study can be found in online repositories. The names of the repository/repositories and accession number(s) can be found in the article/Supplementary Material.

## Author Contributions

EP was responsible for data curation, writing and preparation of the original draft, review and editing of the manuscript, and supervision. KA, CD, IC, GC, and VS was responsible for formal analysis and data curation. BG was responsible for the conceptualization of the study. MG was responsible for the conceptualization of the study, resources, and funding acquisition. RD was responsible for the conceptualization of the study, resources, writing and preparation of the original draft, review and editing of the manuscript, supervision, and funding acquisition. All authors have read and agreed to the published version of the manuscript.

## Conflict of Interest

BG was employed by company Puratos srl. The remaining authors declare that the research was conducted in the absence of any commercial or financial relationships that could be construed as a potential conflict of interest. The reviewer MG declared a past co-authorship with one of the authors, MG, to the handling editor.

## Footnotes

1 http://www.ncbi.nlm.nih.gov/GenBank/

2 https://github.com/yhwu/idemp/blob/master/idemp.cpp

3 https://github.com/qiime2/q2-feature-classifier
