# Supplementary material for: Commercial Organic Versus Conventional Whole Rye and Wheat Flours for Making Sourdough Bread: Safety, Nutritional, and Sensory Implications
Source: Front Microbiol. 2021 Jul 12;12:674413. doi: 10.3389/fmicb.2021.674413 (PMC8312275; doi:10.3389/fmicb.2021.674413)
Supplement: Supplementary file 1 [file Data_Sheet_1.PDF]

Supplementary Material

Supplementary Table 1. Number of sequences analyzed before and after filtering, observed operation taxonomic units (OTUs), Chao1 and Shannon indices for 16S rRNA and ITS amplification from doughs and sourdoughs[a].

Sample ID	N° reads before quality filtering		N° reads after quality filtering		Observed OTUs		Chao1 index		Shannon index	
	16S	ITS	16S	ITS	16S	ITS	16S	ITS	16S	ITS
oSW0	143909	123769	121051	103002	1122	43	1177	43	7.370	2.236
oSW1	129257	99737	109944	82781	896	51	940	53	7.145	1.836
oSW2	131954	124601	111075	100738	1009	72	1068	77	7.244	2.535
oSW5	91878	101675	78903	86258	724	43	807	46	6.606	0.992
oSW10	107091	116109	94467	98681	488	50	514	53	5.131	1.149
cSW0	150231	128816	125666	101007	1082	60	1138	60	7.404	2.921
cSW1	123095	104521	105400	88700	815	39	857	42	7.094	1.579
cSW2	160424	142810	134889	111325	898	75	927	79	7.133	3.107
cSW5	108271	102547	93398	85757	862	70	918	72	6.729	1.835
cSW10	124669	113514	109898	96977	620	44	664	45	5.567	0.875
oR0	273948	143612	226312	111270	1709	91	1747	91	8.076	4.1
oR1	141764	80064	120497	67923	835	47	865	48	7.136	1.254
oR2	105420	120950	88748	93806	751	74	794	74	7.030	3.33
oR5	112197	89654	96696	75060	782	55	827	57	6.370	2.09
oR10	109481	99651	96224	85464	446	39	495	45	5.068	0.691
cR0	265444	144954	215145	111755	1607	143	1675	156	8.297	3.497
cR1	123756	88201	106058	75841	805	30	864	30	7.114	1.175
cR2	107654	117004	91340	90369	988	80	1070	83	7.222	2.984
cR5	100538	143105	86457	119022	871	68	935	70	6.252	1.558
cR10	109716	104801	97099	90099	416	38	446	44	4.941	0.664
oDW0	179416	156617	149145	121810	1186	142	1268	151	7.407	3.53
oDW1	142329	96198	120864	80992	903	59	945	59	7.170	1.831
oDW2	89968	113493	76361	94472	703	84	740	85	7.039	2.3

oDW5	76487	89041	62094	74928	589	45	626	45	6.204	1.507
oDW10	126143	108638	111875	93580	366	26	392	27	4.948	0.449
cDW0	200817	105127	173886	85372	1053	56	1106	57	6.868	2.656
cDW1	120361	90479	103499	78000	790	20	821	20	7.159	0.577
cDW2	96436	93982	82647	78531	758	68	817	72	6.906	1.715
cDW5	69217	89663	59763	75743	643	61	697	62	6.469	1.036
cDW10	124698	137471	110285	114209	530	56	571	58	5.148	1.649

[a]oSW (organic soft wheat), cSW (conventional soft wheat), oR (organic rye), cR (conventional rye), oDW (organic durum wheat), cDW (conventional durum wheat). Doughs prior the first fermentation: oSW0, cSW0, oR0, cR0, oDW0, cDW0; sourdoughs after 1st fermentation: oSW1, cSW1, oR1, cR1, oDW1, cDW1; sourdoughs after 1st refreshment: oSW2, cSW2, oR2, cR2, oDW2, cDW2; sourdoughs after 5th refreshment oSW5, cSW5, oR5, cR5, oDW5, cDW5; sourdoughs after 10th refreshment: oSW10, cSW10, oR10, cR10, oDW10, cDW10. Further details are included in Materials and methods.

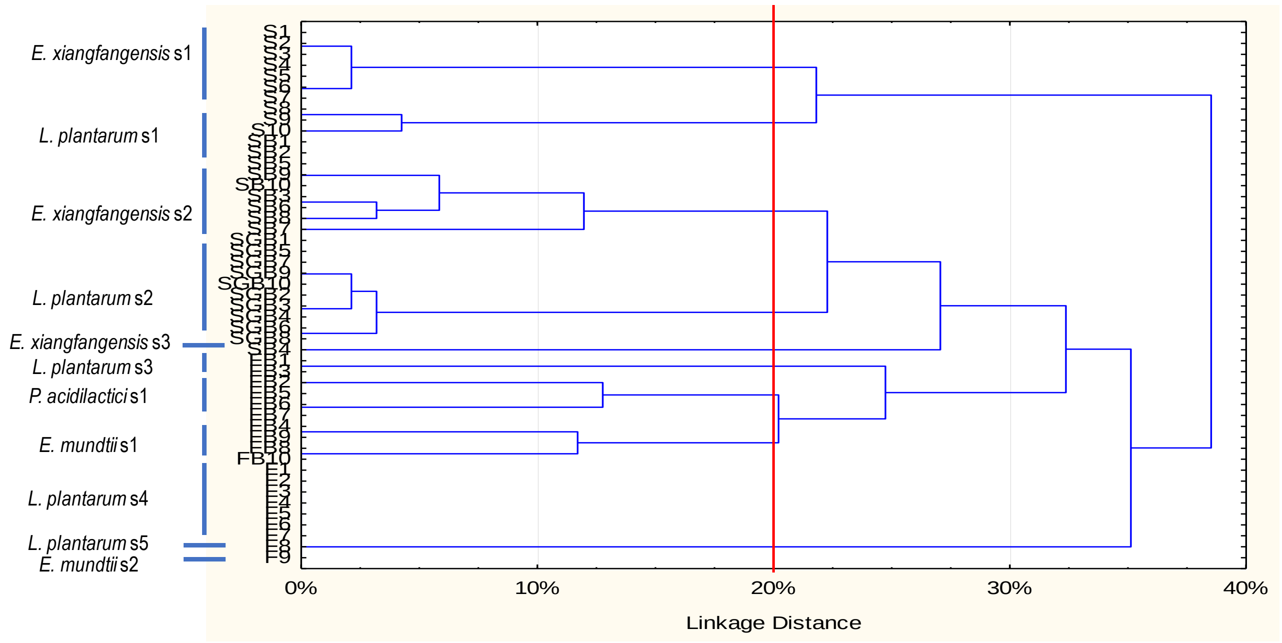

Supplementary Figure 1. Dendrogram of combined (primers P4, P7 and M13) RAPD profiles of lactic acid bacteria strains isolated from cR, cSW, cDW, oR, oSW and oDW flours sourdoughs. Cluster analysis was based on the simple matching coefficient and unweighted pair group method using arithmetic average (UPGMA).

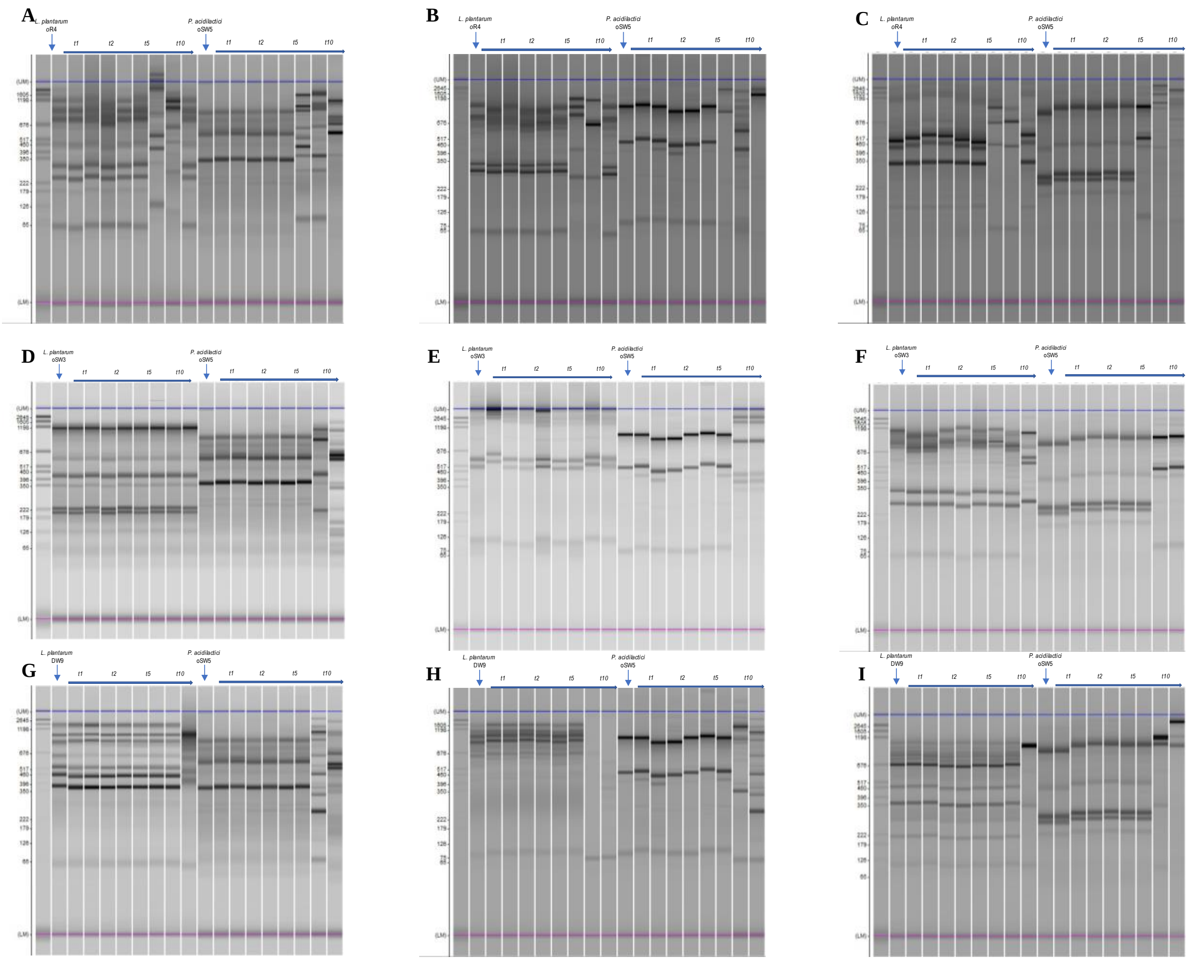

Supplementary Figure 2. Representative randomly amplified polymorphic DNA-PCR (RAPD-PCR) profiles of lactic acid bacteria isolated from the sourdoughs. *Lactiplantibacillus plantarum* oR4, SW3 or DW9 strains were used as binary starters in association with *Pediococcus acidilactici* oSW5 to ferment conventional or organic rye (panels A-C) (oR4 and oSW5), soft (panels D-F) (oSW3 and oSW5) and durum wheat (panels G-I) (DW9 and oSW5) flours depending on the source of isolation of *L. plantarum* strains. *Saccharomyces cerevisiae* N2 was used as yeast in all samples. Bacteria and yeast were inoculated at final cell density of *circa* 7 and 6 log10 cfu/g, respectively. First fermentation (t1) was carried out at 25 °C for 24 h and propagation, through back-slopping (25 °C for 5 h), lasted 10 days. Primers M13 (A, D, G), P4 (B, E, H), and P7 (C, F, I) were used for RAPD-PCR analysis. A pGEM(R) DNA marker (36–2645 bp) was used as molecular size standard. Capillary electrophoretic profiles were singly acquired by MultiNA. Inoculated strains are indicated by arrows.

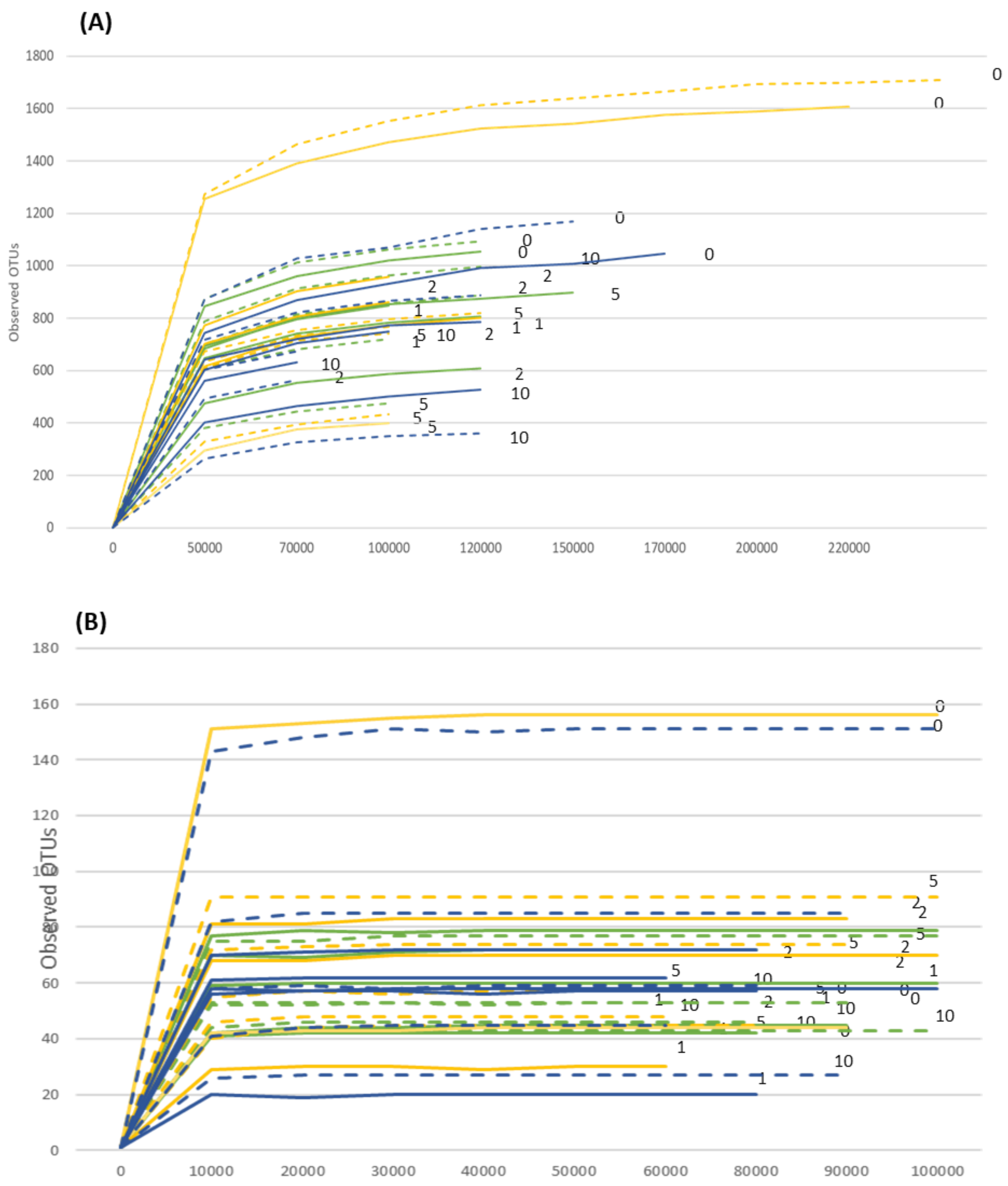
(A)
Observed OTUs
1800
1600
1400
1200
1000
800
600
400
200
0
0
50000
70000
100000
120000
150000
170000
200000
220000
(B)
Observed OTUs
180
160
140
120
60
40
20
0
0
10000
20000
30000
40000
50000
60000
80000
90000
100000

Supplementary Figure 3. Rarefaction curves obtained by QIIME 2.0 for DNA bacterial (A) and yeast (B) samples directly from conventional (c: continuous line) and organic (o: dot line) soft wheat (SW: green), durum wheat (DW: blue) and rye (R: yellow) doughs before (cSW0, oSW0, cDW0, oDW0, cR0, oR0) and after the first fermentation (cSW1, oSW1, cDW1, oDW1, cR1, oR1), and after 1 (cSW2, oSW2, cDW2, oDW2, cR2, oR2), 5 (cSW5, oSW5, cDW5, oDW5, cR5, oR5), and 10 (cSW10, oSW10, cDW10, oDW10, cR10, oR10) days of propagation. Numbers correspond to the fermentation time.

(A)

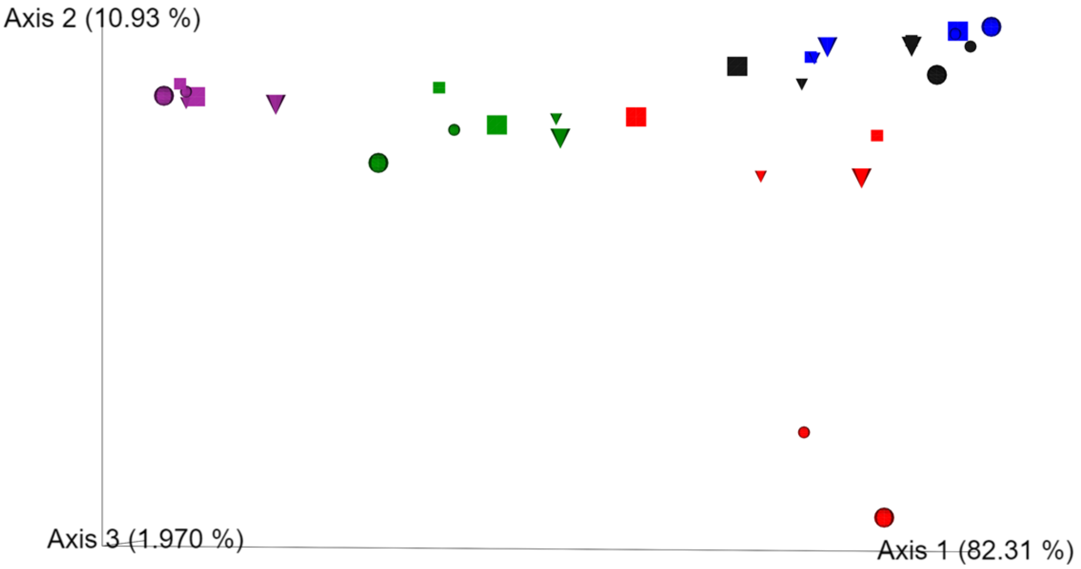

(B)

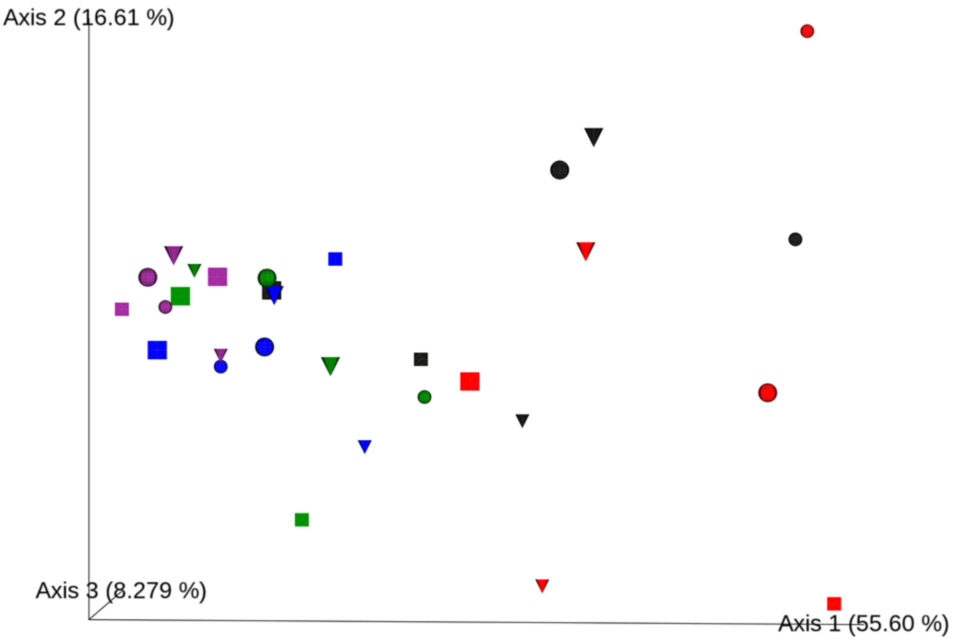

Supplementary Figure 4. Principal coordinate analysis (PCoA) based on Bray Curtis distance analysis of 16S RNA gene (A) and ITS (B) sequences of doughs (before fermentation, red) and sourdoughs (after the 1st fermentation, blue, 1 day of propagation, black, 5 days of propagation, green, and 10 days of propagation, purple) from organic (o) and conventional (c) soft wheat (SW, cone), rye (R, circle) and durum wheat (DW, square). Big and small size symbols represent conventional and organic samples, respectively. Further details are included in Materials and methods.

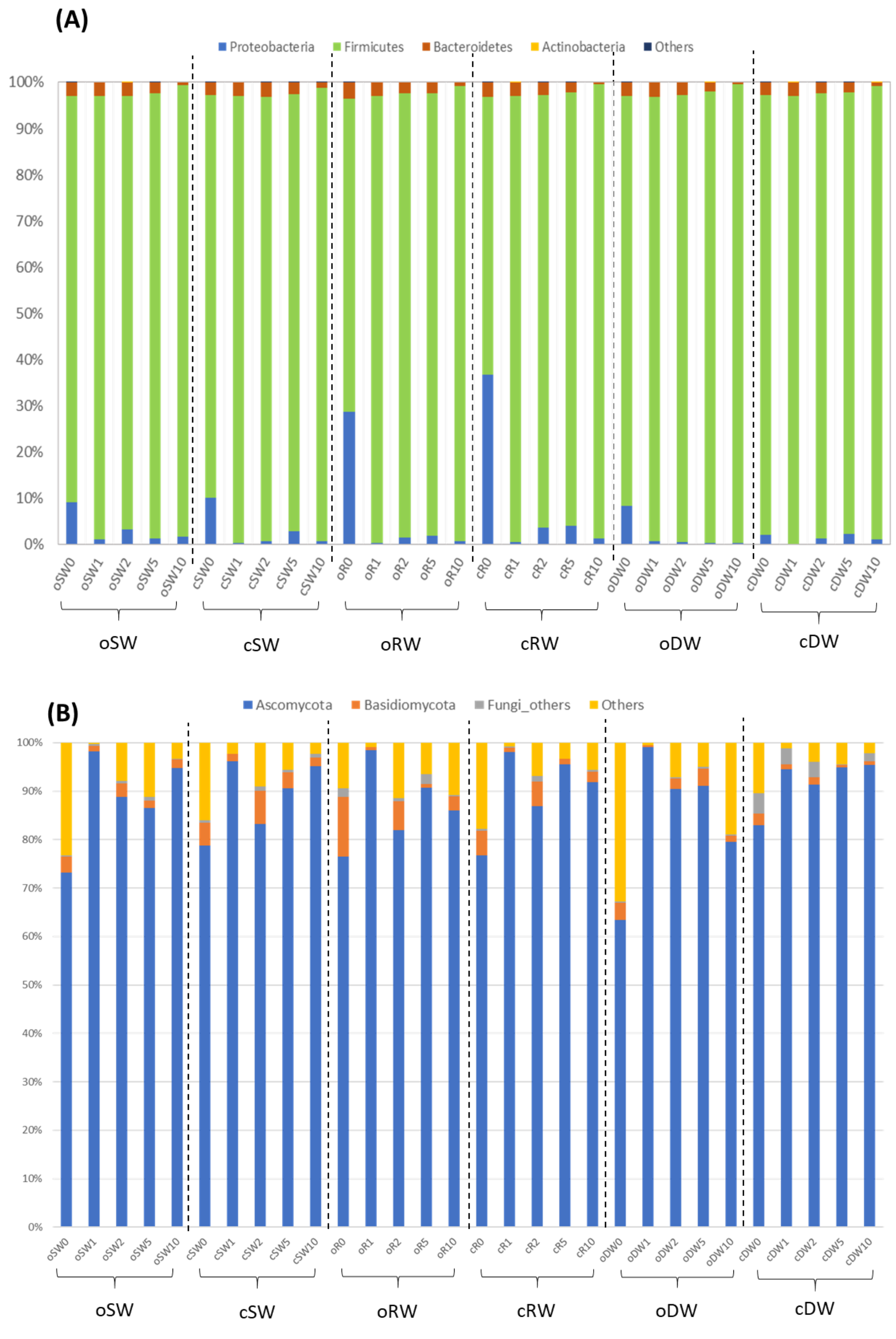

Supplementary Figure 5. Relative abundance of bacterial (A) and yeast (B) phyla in DNA samples directly extracted from conventional (c) and organic (o) soft wheat (SW), durum wheat (DW) and rye (R) doughs before fermentation (cSW0, oSW0, cDW0, oDW0, cR0, oR0), and sourdough after the 1st fermentation (cSW1, oSW1, cDW1, oDW1, cR1, oR1), and after the 1st (cSW2, oSW2, cDW2, oDW2,

cR2, oR2), 5th (cSW5, oSW5, cDW5, oDW5, cR5, oR5), and 10th (cSW10, oSW10, cDW10, oDW10, cR10, oR10) refreshment.
